# Supplementary material for: Integrated energy storage and CO2 conversion using an aqueous battery with tamed asymmetric reactions
Source: Nat Commun. 2024 Feb 1;15:977. doi: 10.1038/s41467-023-44283-y (PMC10834454; doi:10.1038/s41467-023-44283-y)
Supplement: Supplementary file 1 — Supplementary Information [file 41467_2023_44283_MOESM1_ESM.pdf]

Supplementary Information

Integrated energy storage and CO_2 conversion using an aqueous battery with tamed asymmetric reactions

Yumei Liu[1], Yun An[1], Jiexin Zhu[1,2], Lujun Zhu[1], Xiaomei Li[3], Peng Gao[3], Guanjie He[2], Quanquan Pang[1,*]

[1]*Beijing Key Laboratory for Theory and Technology of Advanced Battery Materials, School of Materials Science and Engineering, Peking University, Beijing 100871, China*

[2] *Christopher Ingold Laboratory, Department of Chemistry, University College London, London, WC1H 0AJ, UK*

[3]*International Centre for Quantum Materials, Collaborative Innovation Centre of Quantum Matter, Peking University, Beijing, China*

* E-mail: qqpang@pku.edu.cn

Contents

Supplementary Figures 1-36

Supplementary Tables 1-7

Supplementary Notes 1-19

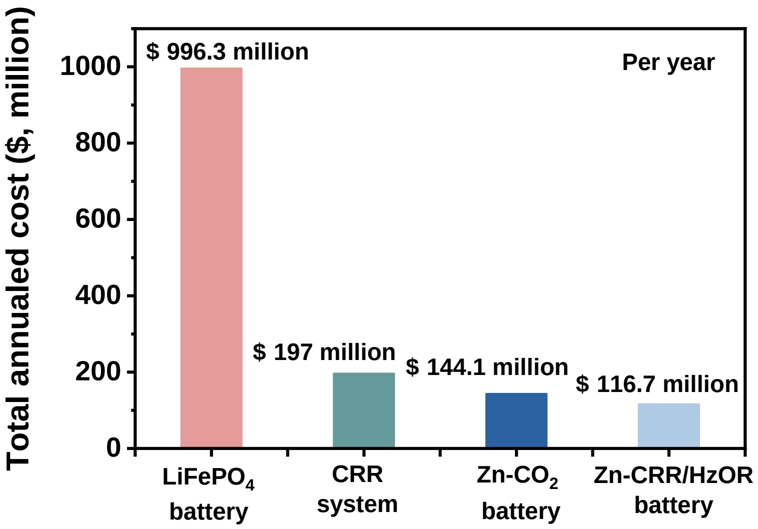

Supplementary Figure 1. A preliminary techno-economic assessment for the Zn-CRR/HzOR, Zn-CO_2, and $LiFePO_4$ batteries, as well as the CRR system.

Supplementary Note 1: Based on the conceptual design, a preliminary techno-economic assessment for the total annualized cost (TAC) of the proposed Zn-CRR/HzOR, the Zn-CO_2 battery, the classical $LiFePO_4$ battery, and the electrochemical CRR system were conducted. Apparently, the Zn-CRR/HzOR is economically favorable among the four technologies due to the least investment and maximum potential profits. Specifically, the minimum total annualized cost (TAC) implies the high potential of profitability, which includes the fixed capital cost, investment cost, and the annual operating cost of the selected system. Our Zn-CRR/HzOR device could fulfill the multiple functions of energy storage, carbon emission reduction, valuable chemical production, and wastewater treatment with the least TAC of 116.7 million $/year, greatly advantageous than other systems ($LiFePO_4$ battery, the stand-alone CRR system, the conventional Zn-CO_2 battery: 996.3, 197, 144.1 million $/year respectively).

The evaluations of $LiFePO_4$ battery and CRR system (CO_2 reduction to CO) are based on the reported data in the previous techno-economic analysis[1,2]. The $LiFePO_4$ battery with 10 tons of cathode material per year was used as the reference and its TAC is the highest among the four systems. We then evaluated the Zn-CO_2 and Zn-CRR/HzOR based on the CRR systems because they shared the similar device system and subsystem components. Thus, their capital and maintenance costs are theoretically

close to each other, except for energy input.

The electricity consumption and price account for a large portion of the total annualized costs for the assessment of CRR system and Zn-CO_2 battery, and the much lower charging voltage greatly reduces the electricity consumption and thus lower the cost for the Zn-CRR/HzOR battery. Herein, an average value of 0.06 \$ kWh is used as the electricity price due to its wide range (0.02 − 0.10 \$ kWh^{-1})[3]. Compared with the CRR system and Zn-CO_2 battery, the less electricity consumption is critical for the least investment of the Zn-CRR/HzOR battery. In addition, another function (hydrazine-containing wastewater treatment) of the Zn-CRR/HzOR battery further decreases the cost, and is also meaningful for the industrial production and environmental protection. Note that we use the reported price data of hydrazine-containing wastewater treatment[4] with an exchange rate of 6.8074 for USD *vs.* RMB (10th February, 2023).

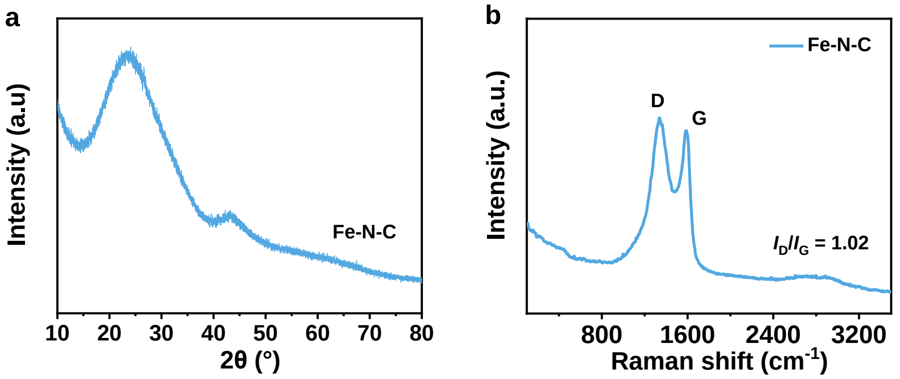

Supplementary Figure 2. Analyses of the Fe-N-C catalyst structure. XRD pattern (a) and Raman spectrum (b) show the carbon structure of Fe-N-C catalyst without Fe-containing crystalline species.

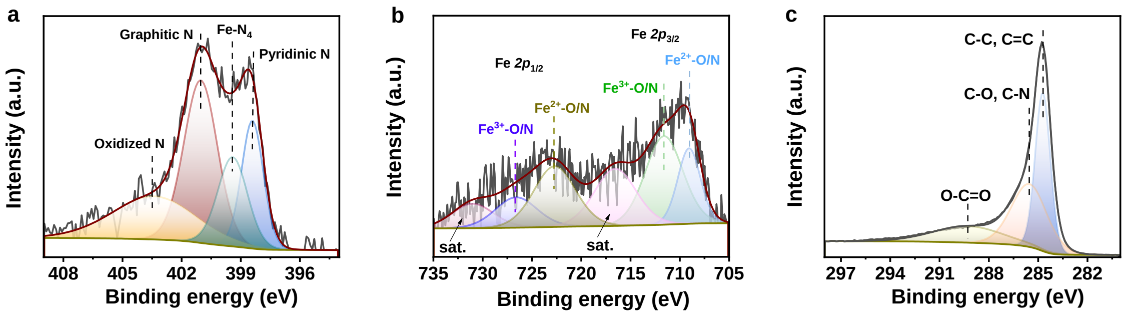

Supplementary Figure 3. XPS measurement of the Fe-N-C catalyst. Fitted XPS spectra of N *1s* (a) Fe *2p* (b), and C *1s* (c) demonstrate that Fe-N-C catalyst consists of elements N, Fe, and C, and the N *1s* and Fe *2p* spectra respectively indicate the possible FeN_4 configuration (a) and the Fe valence (b, between +2 and +3) in the Fe-N-C catalyst.

Supplementary Table 1. Element quantification of Fe-N-C by ICP-OES elemental analysis.

Element (wt%)	Fe	N	C
Fe-N-C	0.70	3.94	78.91

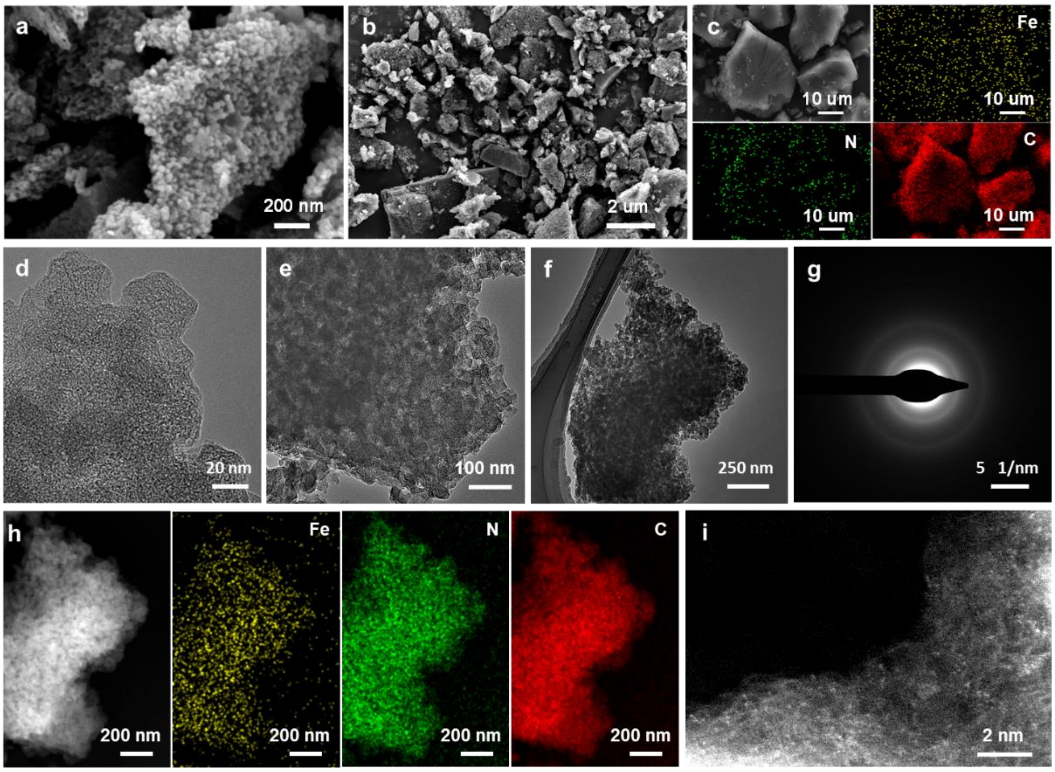

Supplementary Figure 4. Microstructure morphology analysis of the catalyst. SEM and the corresponding EDX elemental mapping images (a-c) showing the nanoparticles morphology and uniform element distribution of the as-prepared Fe-N-C catalyst. Further, its microstructure is also shown by TEM images at different magnifications (d-f). (g) SAED pattern indicating the amorphous structure in Fe-N-C catalyst. (h) STEM-EDX elemental mapping images further implying the uniform distribution of elements Fe, N, and C. (i) Aberration-corrected HAADF-STEM image that demonstrates the atomically dispersed Fe sites in Fe-N-C catalyst.

Supplementary Note 2: The Fe-N-C catalyst was prepared *via* thermolysis process from the ZIF-8 precursor (detailed in the Method section). The X-ray diffraction (XRD) pattern presents two broaden peaks of carbon structure and no Fe-containing crystalline species are observed (**Supplementary Figure 2a**). Raman spectrum of the catalyst shows two *D* and *G* bands (**Supplementary Figure 2b**), further confirming its graphitic carbon structure. The X-ray photoelectron spectroscopy (XPS) studies indicate the presence of FeN_4 configuration (**Supplementary Figure 3**)[5]. The content of Fe in Fe-N-C is quantified to be 0.7 wt% by inductively coupled plasma optical emission spectrometer (ICP-OES, **Supplementary Table 1**). The scanning electron microscope (SEM) and transmission electron microscopy (TEM) imaging studies show that the catalyst is present as nanoparticles (**Supplementary Figure 4**). A ring-like selected area

electron diffraction (SAED) pattern in **Supplementary Figure 4g** reveals an amorphous structure, in accordance with the XRD results. The atomic dispersion of Fe species on the carbon skeleton of Fe-N-C is proven by X-ray spectroscopy (EDS) mapping and the high angle annular dark-field scanning TEM (HAADF-STEM) imaging (**Supplementary Figure 4c, h, i**). The above studies demonstrate the successful construction of single-atom Fe sites in Fe-N-C catalyst.

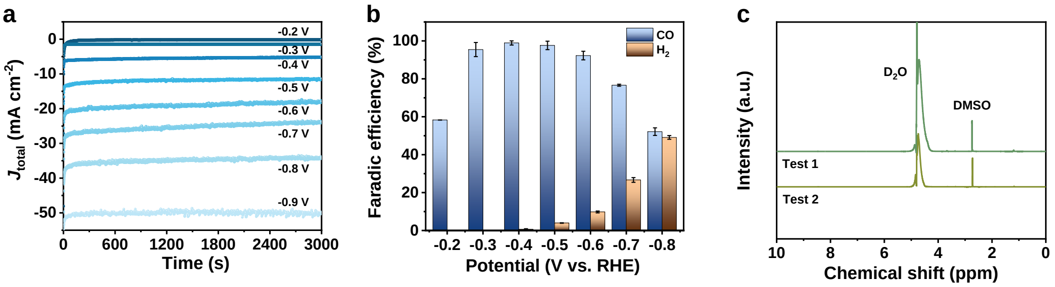

Supplementary Figure 5. Electrochemical CO_2 reduction measurements in the H-type cell. Current responses in CO_2-saturated electrolyte at various potentials from −0.2 to −0.9 V (a) and their corresponding Faradaic efficiencies for conversion to CO and H_2 (b) by using the Fe-N-C catalyst, demonstrating its high selectivity toward CO generation. The error bars in (b) are the standard deviations from three measurements. The ^{1}H NMR spectra of the electrolytes after unidirectional CRR from 0.2 V to 0.8 V confirming no liquid reduction product detected (c).

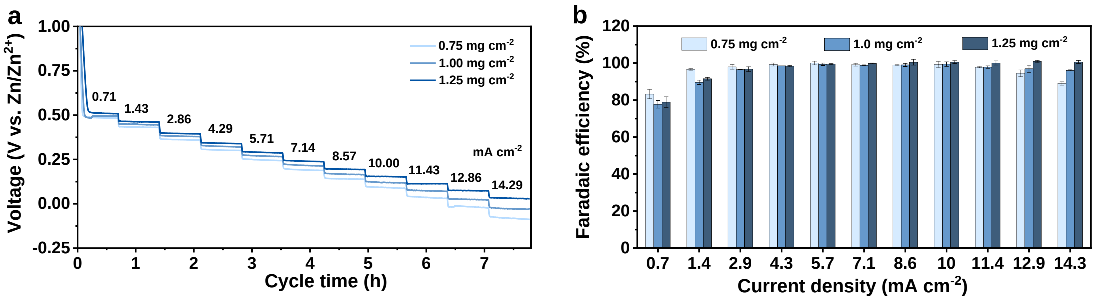

Supplementary Figure 6. Aqueous battery assembled based on different loading mass of cathodes. (a) Discharge voltage against current in the assembled battery is positively related to the loading mass of Fe-N-C catalyst, and similar Faradaic efficiencies of CO generation upon discharging were obtained among the used loading masses (b). The error bars in (b) are the standard deviations from three measurements.

Supplementary Note 3: We first assembled aqueous Zn-CRR/RMOR batteries with

different loading mass (0.75, 1.0, and 1.25 mg cm^{-2}) of Fe-N-C catalyst on the cathodes. In a wide range of current density (0.71 ~ 14.29 mA cm^{-2}), a high FE_{CO} > 98% can be obtained, and there is no significant correlation between FE_{CO} and loading mass (**Supplementary Figure 6a, b**). However, the output voltage shows slight increase with the increasing mass of catalyst (**Supplementary Figure 6a**). For an objective and effective evaluation, the loading mass in this study is established to be 1 mg cm^{-2}. A discharge voltage of 0.5 V and a maximum FE_{CO} as high as 98% in a current density ranging from 0.71 to 12.86 mA cm^{-2} were obtained in the Zn-CRR/RMOR battery, implying that our configuration can efficiently convert CO_2 to CO.

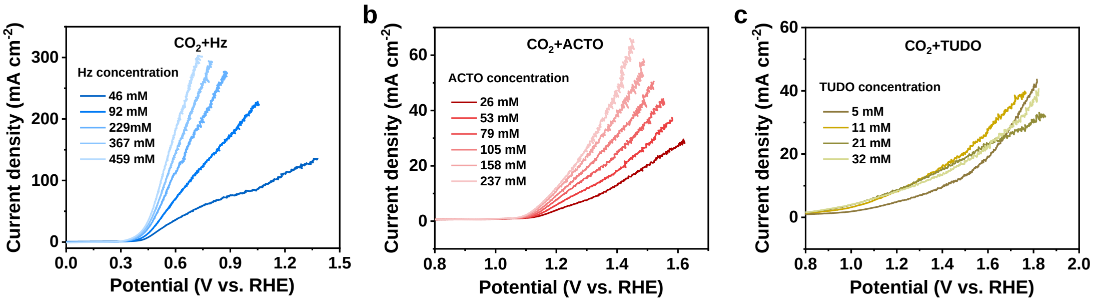

Supplementary Figure 7. The current response-oxidation potential performances as a function of the concentrations for reducing molecules. The LSV curves including HzOR (a), ACTOR (b), and TUDOR (c) show a decreased overpotential with increasing concentration of reducing molecules, and the oxidation potential HzOR is lower than that of ACTOR and TUDOR.

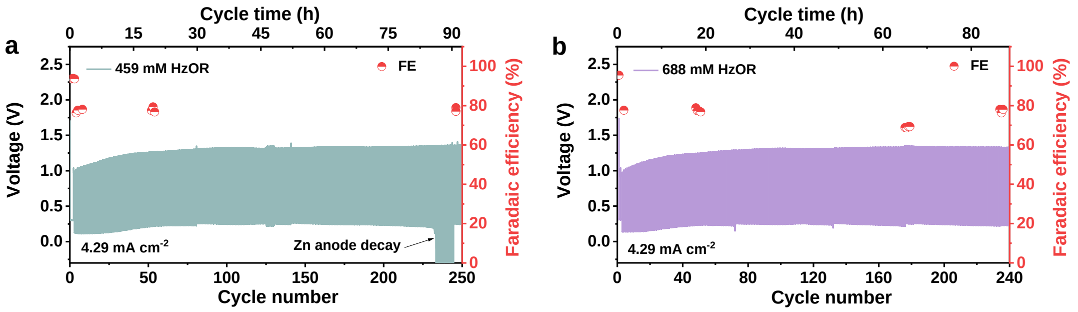

Supplementary Figure 8. The impact of higher concentrations of Hz on voltage and FE_{CO}. The charging voltage of Zn-CRR/HzOR batteries with 459 mM Hz (a) and 688 mM Hz (b).

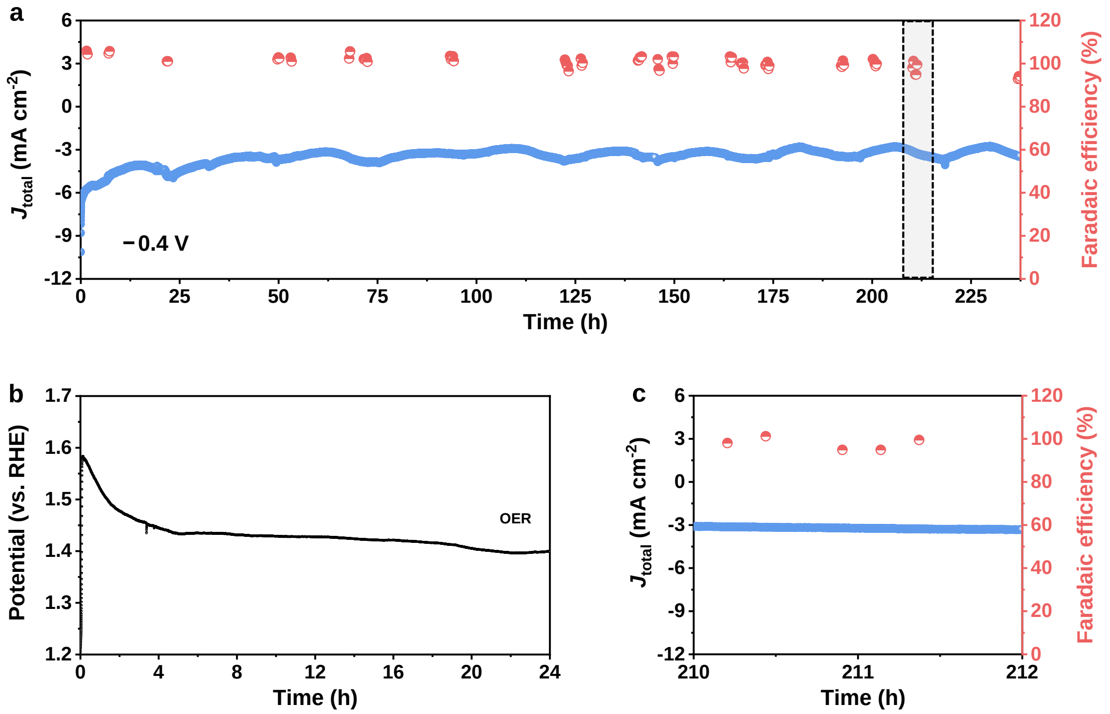

Supplementary Figure 9. Bifunctional electrocatalysis of the catalyst respectively towards CO_2 reduction and oxygen evolution. (a) Excellent long-term stability and high selectivity for conversion to CO were obtained at −0.4 V *vs*. RHE during CRR process. (b) Potential-time response shows a relatively stable OER performance at 4.29 mA cm^{-2}. (c) The enlarged plot of the marked area in (a) displays a clearer view of FE_{CO} evolution from 210 to 212 h.

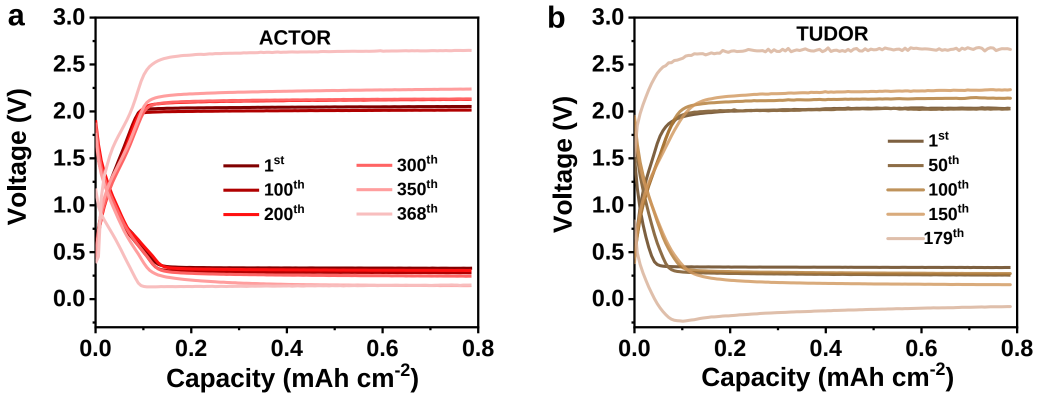

Supplementary Figure 10. Galvanostatic discharge/charge profiles over cycling in Zn-CRR/RMOR batteries. A slight increased voltage polarization over cycling is illustrated in both Zn-CRR/ACTOR battery (a) and Zn-CRR/TUDOR battery (b).

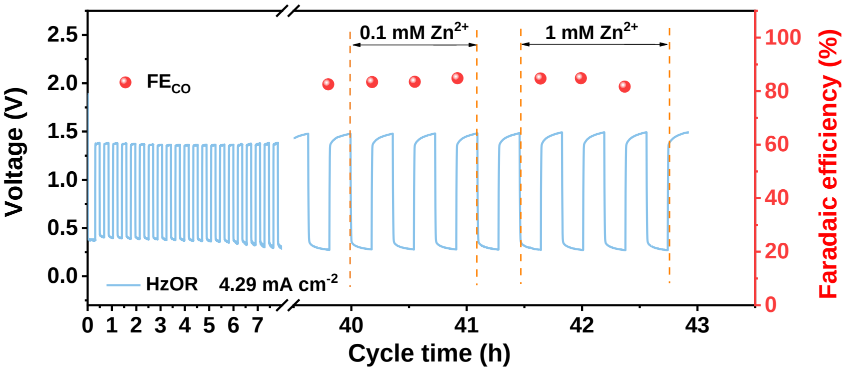

Supplementary Figure 11. The continued stable FE_{CO} and voltage of the Zn-CRR/HzOR battery before and after adding Zn^{2+} (0.1 and 1 mM) into catholyte, demonstrating almost no impact on the electrochemical performance even with Zn^{2+} up to 1 mM.

Supplementary Note 4: For the possible cross-over of Zn^{2+} (by random diffusion for example), we performed an additional experiment to evaluate the impact of the presence of Zn^{2+} on the catholyte (**Supplementary Figure 11**). With 0.1 and 1 mM of Zn^{2+} added to the catholyte chamber, we did not visually observe any solid precipitates in the chamber, and more importantly, the FE_{CO} and voltage of the Zn-CRR/HzOR battery remained stable before and after adding Zn^{2+} (0.1 and 1 mM) to the catholyte, confirming that Zn^{2+} has no noticeable effect on our Zn-CRR/HzOR battery.

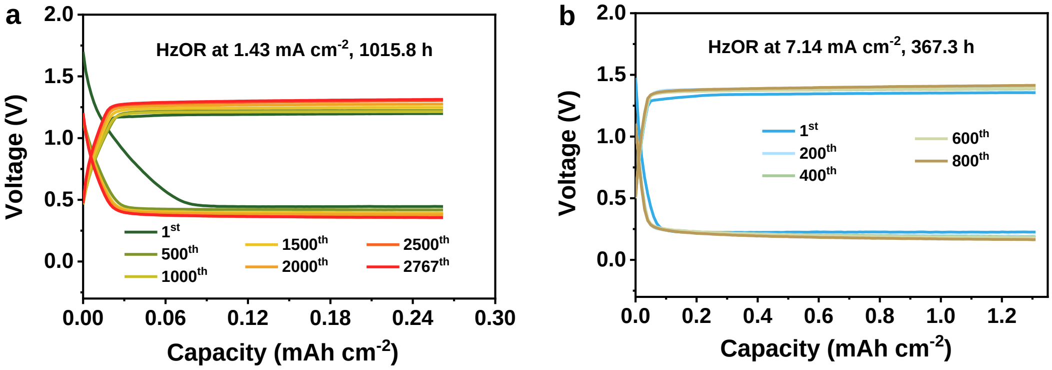

Supplementary Figure 12. The voltage polarization over long-term cycling of Zn-CRR/HzOR battery. Marginal increase in the voltage polarization over long-term cycling is observed at both current densities, *i.e.,* 1.43 (a) and 7.14 (b) mA cm^{-2}.

Supplementary Table 2. Outstanding electrochemical performance obtained in our Zn-CRR/HzOR batteries compared to the reported Zn-CO_2 batteries. As the potential upon OER is 1.23 V, and that for the Zn^{2+}→Zn is –0.76 V, we note that the charging voltage is theoretically about 2 V. For those with charging voltage lower than 2 V, we speculate that undesired side reactions (such as oxidation of some unstable substances) may have occurred rather than OER.

	**Cathode catalyst**	**Catholyte; Anolyte**	**Maximum FE**	**Discharging voltage**	**Charging voltage**	**Cycling stability**	**Ref**
1	Fe-N-C	0.5 M $KHCO_3$;<br>1 M KOH + 0.02 M $Zn(CH_3COO)_2$	98.5%, 2.86 ~ 14.29 mA cm^{-2}	0.50 V, 0.71 mA cm^{-2}<br>0.40 V, 2.86 mA cm^{-2}<br>0.23 V, 7.14 mA cm^{-2}<br>0.1 V, 11.43 mA cm^{-2}	1.16 V, 0.71 mA cm^{-2}<br>1.26 V, 2.86 mA cm^{-2}<br>1.40 V, 7.14 mA cm^{-2}<br>1.52 V, 11.43 mA cm^{-2}	2768 cycles (1015.8), 1.43 mA cm^{-2}, 1140 cycles (418 h), 4.29 mA cm^{-2}, 850 cycles (311.7 h), 7.14 mA cm^{-2}	This work
2	Ni-N_3-C	0.8 M $KHCO_3$;<br>0.8 M KOH + 0.02 M $Zn(CH_3COO)_2$	93%, 2 mA	0.41 V, 0.5 mA	2.4 V, 0.5 mA	100 cycles, 2 mA	6
3	s-SnLi alloy	0.1 M $KHCO_3$;<br>6 M KOH + 0.02 M $Zn(CH_3COO)_2$	-	0.6 V, 0.5 mA cm^{-2}<br>0.14 V, 8.8 mA cm^{-2}	~2.3 V, 0.5 mA cm^{-2}	800 cycles (85 h)<br>0.5 mA cm^{-2}	7
4	CA/N-Ni aerogel	0.5 M $KHCO_3$;<br>6 M KOH + 0.2 M $Zn(CH_3COO)_2$	98%, ~2.5 mA cm^{-2}	~0.32 V, 0.5 mA cm^{-2}<br>~0.13 V, 3 mA cm^{-2}	~2.7 V, 0.5 mA cm^{-2}	90 cycles (30 h)<br>0.5 mA cm^{-2}	8
5	Coralloid Au	0.5 M $KHCO_3$;<br>6 M KOH + 0.2 M $Zn(CH_3COO)_2$	63%, 3.0 mA cm^{-2}	0.45 V, 1.0 mA cm^{-2}<br>0.2 V, 3.0 mA cm^{-2}	2.8 V, 1.0 mA cm^{-2}	68 h, 1.0 mA cm^{-2}	9
6	Ni–N_x 2D/NPC	1 M $KHCO_3$;<br>1 M KOH + 0.02 M $Zn(CH_3COO)_2$	95%, 2.5 mA cm^{-2}	0.30 V, 0.5 mA cm^{-2},<br>0.16 V, 1.5 mA cm^{-2}	2.50 V, 0.5 mA cm^{-2}<br>2.98 V, 1.5 mA cm^{-2}	50 h, 0.25 mA cm^{-2}	10
7	Fe_1–Ni_1–N–C	0.8 M $KHCO_3$;<br>0.8 M KOH + 0.02 M $Zn(CH_3COO)_2$	93.4%, 1 mA	~0.25 V, 1.1 mA	~2.25V, 1.1 mA	15 h, 1.1 mA	11
8	NiFe-DASC	2 M KCl;<br>2 M KOH + 0.02 M $Zn(CH_3COO)_2$	90.6%, 5 mA cm^{-2}	0.43, 1.5 mA cm^{-2}<br>0.12 V, 10 mA cm^{-2}	2.16 V, 0.1 mA cm^{-2}<br>3.01 V, 10 mA cm^{-2}	180 cycles (90 h)<br>5 mA cm^{-2}	12
9	HPC-Co/CoPc (5:1)	1 M $KHCO_3$;<br>6 M KOH + 0.2 M $Zn(CH_3COO)_2$	65 %, 10 mA cm^{-2}	0.7 V, 0.5 mA cm^{-2}	2.32 V, 0.5 mA cm^{-2}	130 cycles, 0.5 mA cm^{-2}	13
10	BiC/HCS	0.8 M $KHCO_3$;<br>6 M KOH + 0.2 M $Zn(CH_3COO)_2$	92%, 3 mA	~0.2 V, 1 mA cm^{-2}	~2.25 V, 1 mA cm^{-2}	200 cycles (65 h)<br>1mA cm^{-2}	14

11	Fe-SA/BNC	0.8 M $KHCO_3$; 0.8 M KOH + 0.02 M $Zn(CH_3COO)_2$	98.9%, 1 mA cm^{-2}	~0.51 V, 1 mA cm^{-2}	~2.25 V, 1 mA cm^{-2}	80 cycles (27 h) 1mA cm^{-2}	15
12	Bi nanoparticles	0.5 M $KHCO_3$; 1 M KOH	80%, 8 mA cm^{-2}	~0.3 V, 5 mA cm^{-2}	~2.6 V, 5 mA cm^{-2}	120 cycles (20 h) 5 mA cm^{-2}	16
13	s-PdNi/CNFs-1000	0.1 M $KHCO_3$; 6 M KOH + 0.2 M $Zn(CH_3COO)_2$	~92.6% 3 mA cm^{-2}	0.86 V, 1.2 mA cm^{-2}	~1.5 V, 1.2 mA cm^{-2}	35 h, 1.2 mA cm^{-2}	17
14	Cu–N_2/GN	0.5 M $KHCO_3$; 6 M KOH + 0.2 M $Zn(CH_3COO)_2$	64%, 1.4 mA cm^{-2}	0.7 V, 1 mA cm^{-2}	2.4 V, 1 mA cm^{-2}	120 cycles (40 h) 1 mA cm^{-2}	18
15	CoPc@DNHCS-8	0.8 M $KHCO_3$; 0.8 M KOH + 0.02 M $Zn(CH_3COO)_2$	94%, 3.0 mA cm^{-2}	0.34 V, 3.0 mA cm^{-2}	2.75 V, 3.0 mA cm^{-2}	40 h, 1 mA cm^{-2}	19
16	DNG-SAFe	1 M $KHCO_3$; 6 M KOH + 0.2 M $Zn(CH_3COO)_2$	86.5%, 5 mA cm^{-2}	0.6 V, 0.1 mA	2.23 V, 0.1 mA	150 cycles (50 h) 0.5mA cm^{-2}	20
17	Fe_1NC/S1-1000	0.8 M $KHCO_3$; 0.8 M KOH + 0.02 M $Zn(CH_3COO)_2$	-	~0.7 V, 0.5 mA cm^{-2}	~1.2 V, 0.5 mA cm^{-2}	72 cycles (25 h) 0.5 mA cm^{-2}	21
18	Abundant-defects Bi	2 M $KHCO_3$ + 0.02 M $HCOO^-$; 2 M KOH + 0.02 M $Zn(CH_3COO)_2$	-	~0.35 V, 5 mA cm^{-2}	~3.3 V, 5 mA cm^{-2}	66 cycles (22 h) 5 mA cm^{-2}	22
19	Fe–P Nanocrystals	1 M $KHCO_3$; 6 M KOH + 0.02 M $Zn(CH_3COO)_2$	92%, 0.5 mA cm^{-2}	0.49, 0.34 V, 0.5, 1 mA cm^{-2},	2.33 V, 0.5 mA cm^{-2}	500 cycles (168 h) 0.5 mA cm^{-2}	23
20	ZnTe/ZnO@C	0.8 M $KHCO_3$; 0.8 M KOH + 0.02 M $Zn(CH_3COO)_2$	68%, 5 mA cm^{-2}	0.5 V, 1 mA cm^{-2}	2.2 V, 1 mA cm^{-2}	108 cycles (36 h) 1 mA cm^{-2}	24
21	CuNi-DSA/CNFs	0.1 M $KHCO_3$; 6 M KOH + 0.2 M $Zn(CH_3COO)_2$	98.2%, 3 mA cm^{-2}	~1.1 V, 1.5 mA cm^{-2}	~1.75 V, 1.5 mA cm^{-2}	56 h, 1.5 mA cm^{-2}	25
22	BiO_{2-x} nanosheets	1 M KHCO; 2 M KOH + 0.02 M $Zn(CH_3COO)_2$	-	~0.2 V, 4.5 mA cm^{-2}	~3.3 V, 4.5 mA cm^{-2}	300 cycles (100 h) 4.5 mA cm^{-2}	26
23	Ni-N/OMC-1	0.8 M $KHCO_3$; 0.8 M KOH	92%, 2.5 mA	~0.1 V, 0.5 mA cm^{-2}	~2.5 V, 0.5 mA cm^{-2}	125 cycles (25 h) 0.5 mA	27
24	Ni_1-N-C (Cl)	0.8 M $KHCO_3$; 0.8 M KOH + 0.02 M $Zn(CH_3COO)_2$	93.8%, 2 mA	0.175 V, 2.5 mA	~2.9 V, 2.5 mA	24 h, 2.0 mA cm^{-2}	28

25	Ir@Au	0.8 M $KHCO_3$; 0.8 M KOH + 0.02 M $Zn(CH_3COO)_2$	90%, 1.5 mA	0.59 V, 0.1 mA ~0.25 V, 1.5 mA	~2.25 V, 0.1 mA, ~2.8 V, 1.5 mA	90 cycles (30 h) 0.9 mA	29
26	N-SnO_2 NS	-	74%, 6.0 mA cm^{-2}	0.97 V 1.0 mA cm^{-2}	2.60 V, 1.0 mA cm^{-2}	100 cycles (33 h) 1.0 mA cm^{-2}	30
27	NiPG nanomaterial	3 M $KHCO_3$ +1.5 M KCl; 6 M KOH + 0.2 M $Zn(CH_3COO)_2$	66%, 1.5 mA	0.47 V, 0.25 mA 0.29 V, 1.5 mA	2.58 V, 0.25 mA 2.70 V, 1.5 mA	65 cycles (13 h) 0.5 mA	31
28	Co SAs@NCMF	1 M $KHCO_3$ 6 M KOH + 0.2 M $Zn(CH_3COO)_2$	> 90%, 2–5 mA cm^{-2}	~0.4V, 0.5 mA cm^{-2} ~0.25V, 2 mA cm^{-2}	~2.6 V, 0.5 mA cm^{-2} ~2.82 V, 2 mA cm^{-2}	1000 cycles (166.7 h) 0.5 mA cm^{-2}	32
29	Ni-CNC-100	0.8 M $KHCO_3$; 0.8 M KOH + 0.02 M $Zn(CH_3COO)_2$	~79.6% 2.5 mA cm^{-2}	~0.4 V, 0.5 mA cm^{-2}	~1.5 V, 0.5 mA cm^{-2}	130 cycles (40 h) 0.5 mA cm^{-2}	33
30	Zn/NC NSs	0.5 M $KHCO_3$ 6 M KOH + 0.02 M $Zn(CH_3COO)_2$	95%, 1.5 mA cm^{-2}	~0.37 V, 1.0 mA cm^{-2}	~2.8 V, 1.5 mA cm^{-2}	~27 h, 1.5 mA cm^{-2}	34
31	$Ni_4N/Ni_3ZnC_{0.7}$	0.1 M $KHCO_3$ 6 M KOH + 0.2 M $Zn(CH_3COO)_2$	~68.9%, 4 mA cm^{-2}	~0.45 V, 0.5 mA cm^{-2}	~2.4 V, 0.5 mA cm^{-2}	15 h, 0.5 mA cm^{-2}	35
32	SAs–Ni–N–C	0.5 M $KHCO_3$ 6 M KOH + 0.2 M $Zn(CH_3COO)_2$	93.3%, 2mA cm^{-2}	~0.3 V, 2mA cm^{-2}	~1.35 V, 1.0 mA cm^{-2}	32 h, 1.0 mA cm^{-2}	36
33	$PdCu_3$/NC	0.8 M $KHCO_3$; 0.8 M KOH + 0.02 M $Zn(CH_3COO)_2$	98%%, 0.9mA cm^{-2}	0.44 V, 1.7 mA cm^{-2}	~2.3V, 0.5 mA cm^{-2}	400 cycles (133 h) 0.5mA cm^{-2}	37
34	BiPd/C	0.1 M $KHCO_3$ + 0.1 M HCOONa 1 M KOH + 0.02 M $Zn(CH_3COO)_2$	52.64%, 4mA cm^{-2}	0.12 V, 4 mA cm^{-2}	1.6 V, 0.5 mA cm^{-2}	~45 h, 0.5 mA cm^{-2}	38
35	Sn/NCNFs	0.5 M $KHCO_3$ 6 M KOH + 0.02 M $Zn(CH_3COO)_2$	97.66%, 1.0 mA cm^{-2}	0.28 V, 1.5 mA cm^{-2}	~2.4 V, 0.5 mA cm^{-2}	~36 h, 0.5 mA cm^{-2}	39
36	C-BN@600	1 M $KHCO_3$; 1 M KOH + 0.02 M $Zn(CH_3COO)_2$	50.9%, 1 mA cm^{-2}	~0.5 V, 1 mA cm^{-2}	~2.3 V, 1mA cm^{-2}	~300 h, (800 cycles) 1 mA cm^{-2}	40
37	SnO_2/MXene	0.1 M $KHCO_3$ 1 M KOH + 0.02 M $Zn(CH_3COO)_2$	85%, 5 mA cm^{-2}	0.53 V, 2 mA cm^{-2}	~1.3 V, 2 mA cm^{-2}	60 h, 2 mA cm^{-2}	41
38	Tiny Sn-ene	0.5 M $KHCO_3$	-	~0.55 V, 1mA cm^{-2}	~2.7 V, 1mA cm^{-2}	~100 h, 1 mA	42

	QDs	6 M KOH + 0.02 M $Zn(CH_3COO)_2$				cm^{-2}	
39	Ni-Mn-Cu-Co-Fe-Al oxides	0.5 M $KHCO_3$ 1 M KOH + 0.02 M $Zn(CH_3COO)_2$	-	0.59 V, 0.025 mA cm^{-2}	2.40 V, 0.025 mA cm^{-2}	42 h, 0.025 mA cm^{-2}	43
40	Ni@N-C	1 M $KHCO_3$ 6 M KOH + 0.02 M $Zn(CH_3COO)_2$	90%, 6 mA cm^{-2}	0.5 V, 0.5 mA cm^{-2}	2.5 V, 0.5 mA cm^{-2}	160 cycles (45 h) 0.5 mA cm^{-2}	44
41	Ti–Bi NSs	0.8 M $KHCO_3$ 0.8 M KOH + 0.02 M $Zn(CH_3COO)_2$	81%, 6 mA cm^{-2}	~0. 2 V, 1 mA cm^{-2}	~2.2 V, 1 mA cm^{-2}	54 cycles (27 h) 1 mA cm^{-2}	45
42	Fe/Ni-N-C	0.5 M $KHCO_3$ 6 M KOH + 0.2 M $Zn(CH_3COO)_2$	97.6%, 6 mA cm^{-2}	0.52 V, 1 mA cm^{-2}	>2.5 V, 1.5 mA cm^{-2}	420 cycles (140 h) 1.5 mA cm^{-2}	46
43	Pd1-O-CB	0.5 M $KHCO_3$ 6 M KOH + 0.02 M $Zn(CH_3COO)_2$	-	0.5 V, 1.5 mA cm^{-2}	2 V, 1.5 mA cm^{-2}	100 h, 1.5 mA cm^{-2}	47
44	Si doping-Fe-N-C	1 M $KHCO_3$ 6 M KOH + 0.2 M $Zn(CH_3COO)_2$	94.53%, 10 mA cm^{-2}	0.65V, 0.5 mA cm^{-2}	~1.7 V, 0.5 mA cm^{-2}	<12 h, 0.5 mA cm^{-2}	48
45	Fe@NPC	0.8M $KHCO_3$ 0.8 M KOH + 0.02 M $Zn(CH_3COO)_2$	70.1%, 1 mA	~2 V, 0.5 mA cm^{-2}	~0.5 V, 0.5 mA cm^{-2}	120 cycles (40 h) 0.5 mA cm^{-2}	49

Supplementary Table 3. The charge and discharge products in the Zn-CO_2 and Zn-CRR/MROR batteries.

	Discharge product	Charge product
Zn-CO_2	CO, H_2	O_2
Zn-CRR/HzOR	CO, H_2	N_2
Zn-CRR/ACTOR	CO, H_2, HCOOH	Acetone
Zn-CRR/TUDOR	CO, H_2	SO_4^{2-}, $CO(NH_2)_2$

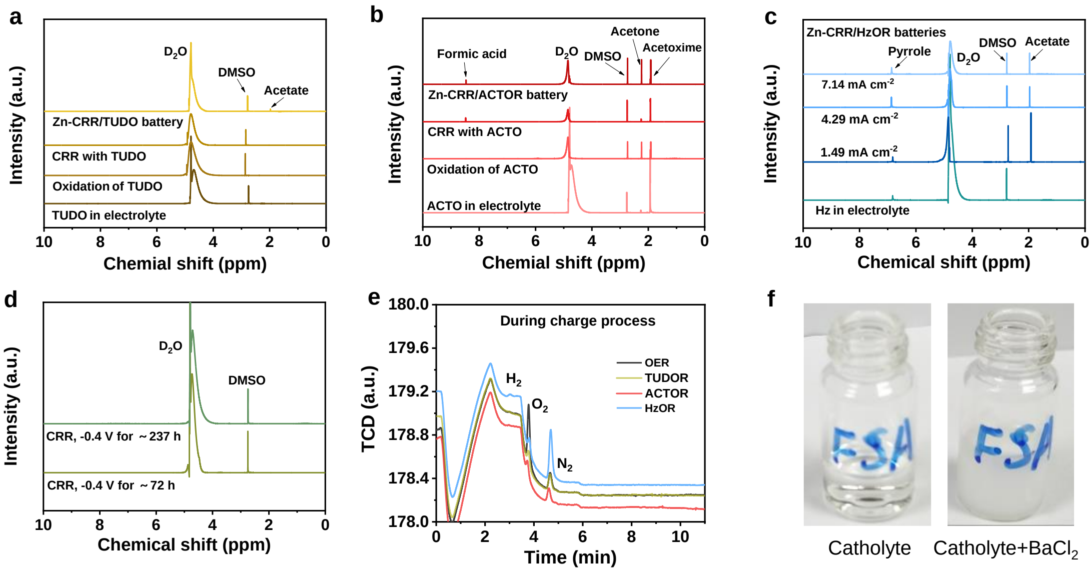

Supplementary Figure 13. Determination of the oxidation products of reducing molecules during charging by online GC and ^{1}H NMR measurements. (a-c) ^{1}H NMR spectra of the electrolytes after oxidation of TUDO/ACTO/Hz, or with the presence of ACTO/TUDO/Hz, or the catholytes in the Zn-CRR/RMOR batteries. The presence of acetate in (a) is due to the acetate ion from anolyte crossing to catholyte after the long-term operation. (d) The ^{1}H NMR spectra of the electrolytes after unidirectional CRR (~72 and ~237 h), demonstrating no liquid reduction product detected. (e) During charging, the signals of thermal conductivity detector (TCD) showing more intensive O_2 and N_2 peaks in the OER and HzOR cases, respectively. Notably, other additional peaks (*i.e.* the residual O_2 and N_2) are common in the GC measurement system. (f) Digital image demonstrating the appearance of the white $BaSO_4$ precipitate after adding $BaCl_2$ into the TUDOR-based catholyte.

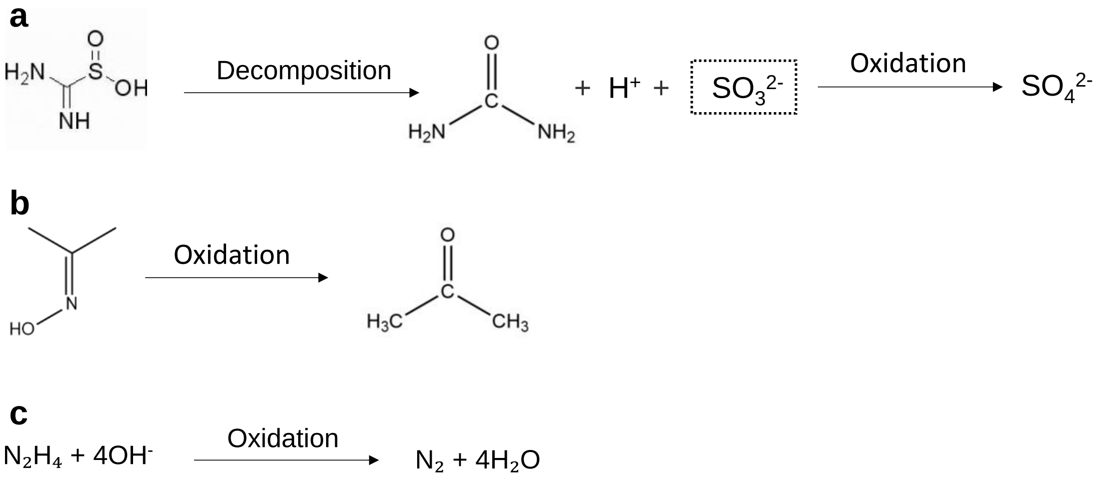

Supplementary Figure 14. Schematic of the electrochemical reaction processes (main oxidation product) corresponding to TUDOR (a), ACTOR (b), and HzOR (c).

Supplementary Note 5: For analyses of the oxidation products, the catholytes were collected for the determination of liquid oxidation products by ^{1}H nuclear magnetic resonance (NMR). Together with the decoupled electrochemical processes in **Supplementary Figure 13**, N_2, acetone, and SO_4^{2-} are demonstrated to be the products of HzOR, ACTOR, and TUDOR, respectively. TUDO and urea contain only the reactive hydrogen atom that is difficult to be detected by ^{1}H NMR spectra in the TUDO-containing systems in addition to the internal standard (dimethyl sulfoxide, DMSO). White precipitate appeared after adding $BaCl_2$ (**Supplementary Figure 13f**) into the catholyte of the Zn-CRR/TUDOR battery, demonstrating the presence of SO_4^{2-}, in according with the schematic reaction process in **Supplementary Figure 14a**. The TUDO molecules in electrolyte first decompose into urea and H_2SO_3, and thus H_2SO_3 serves as the molecule to be reduced. Among the three reducing molecules, the longest cycling life was evidenced in the case of HzOR. This is attributed to the gaseous product N_2 produced by HzOR, which is superior to the carbonaceous organics that can poison the active sites and cause the battery failure[50].

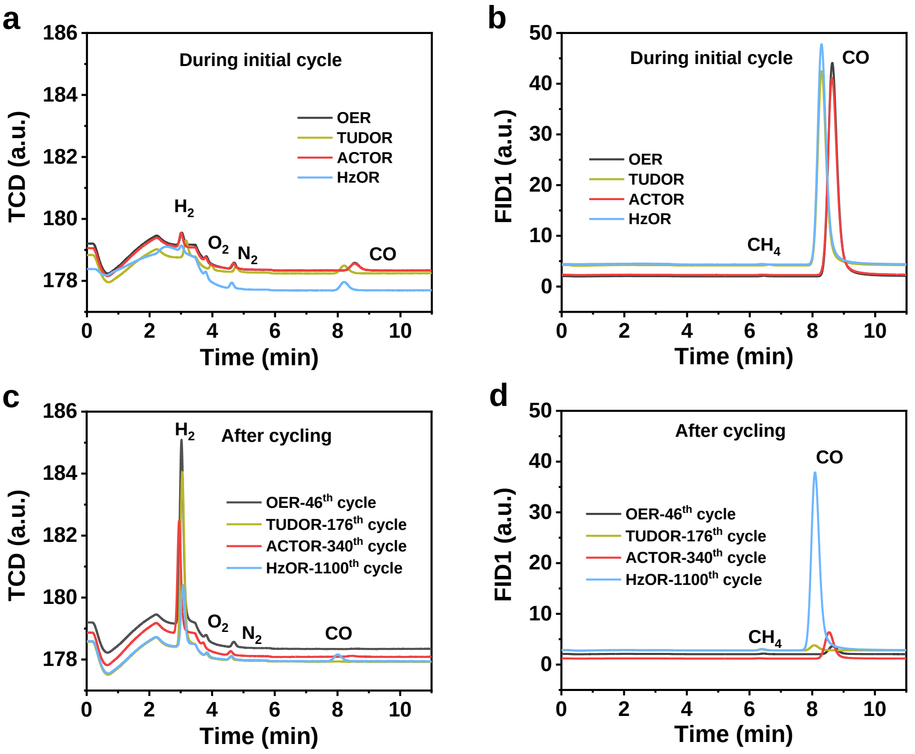

Supplementary Figure 15. GC spectra of the Zn-CRR/RMOR batteries based on HzOR, ACTOR, and TUDOR during discharging process. (a-b) The signals of TCD (a) and FID1 (b) during initial discharge process demonstrate that CO is the dominating product, and indicate the highest CRR activity in Zn-CRR/HzOR battery. (c-d) Compared to OER, TUDOR, and ACTOR, the weaker signals of TCD (c) and stronger FID1 signals (d) after long-term cycling indicate that high CRR activity is retained in the Zn-CRR/HzOR battery. Note that the trace amount of O_2, N_2, CH_4, and CO is from the used CO_2 gas.

Supplementary Note 6: For the discharge process, CO and H_2 are the products of all Zn-CRR/RMOR batteries. Specifically, CO is the main product with a slight amount of H_2, which are confirmed and quantified by GC, as shown in **Supplementary Figure 15**. Another product, HCOOH, was also observed in the case of ACTOR, as evidenced by ^{1}H NMR spectrum in **Supplementary Figure 13b**. It is clear that superior CRR activity after 1100 cycles was achieved in the Zn-CRR/RMOR battery with FE_{CO} as high as 92%. In contrast, H_2 generation with FE (FE_{H_2}) as high as ~ 86% was shown in the OER-based battery, indicating the battery failure. Also, ~ 46% FE_{H_2} was found in the Zn-CRR/ACTOR battery (**Supplementary Figure 15**). This suggests that the remaining current is consumed by another product, in accordance with the observed HCOOH formation evidenced by ^{1}H NMR spectrum in **Supplementary Figure 13b**.

Nevertheless, this indicates the possibility for obtaining high value-added CRR products by taming the oxidation of reducing molecules.

To disprove the possible reduction of TUDO and ACTO, CO_2 were continuously bubbled into the electrolytes. No corresponding products could be detected in these two systems, indicating the CRR is the dominating reaction. Notably, the presence of acetone in the two solutions (the one of "CRR with ACTO" and the one of "ACTO in electrolyte") can be attributed to the oxidation of ACTO exposed to atmosphere (**Supplementary Figure 13b**). Similarly, in the systems containing Hz, the trace amount of pyrrole is the impurity from the used Hz (**Supplementary Figure 13c**).

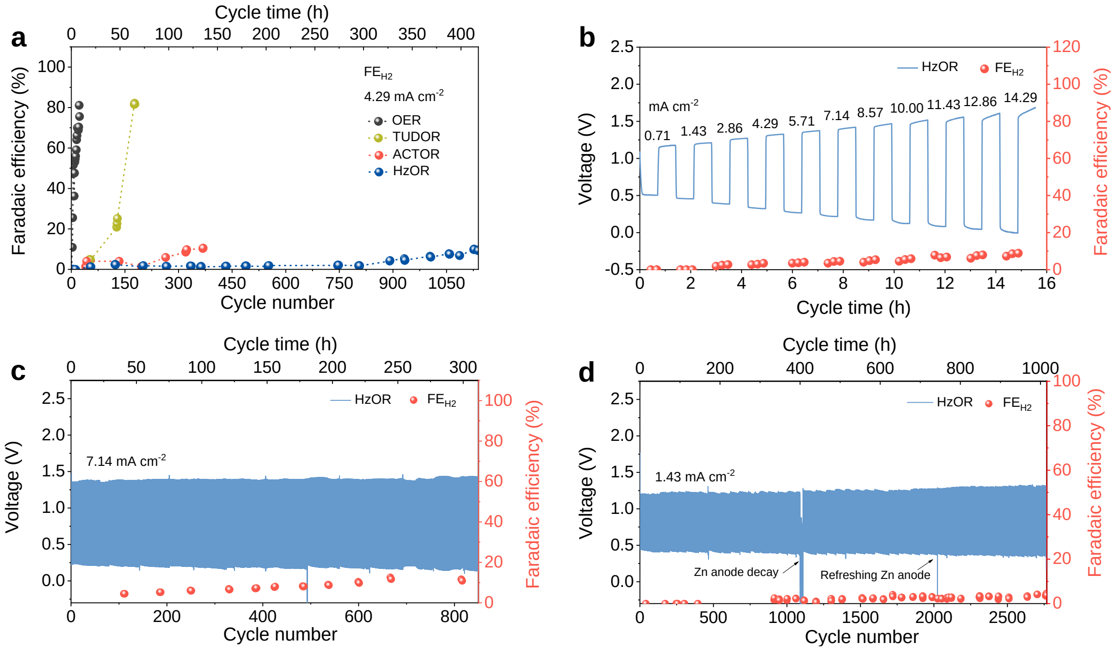

Supplementary Figure 16. Electrochemical performances of the Zn-CRR/RMOR batteries. (a) The corresponding FE_{H_2} of the Zn-CRR/RMOR batteries with varied RMORs at 4.29 mA cm^{-2} (with OER as the reference). (b) The Zn-CRR/HzOR battery showing low FE_{H_2} during the rate capability test (69 mM Hz). (c-d) Long-term cycling performance of the Zn-CRR/HzOR battery demonstrating continued low selectivity for H_2 generation at 7.14 mA cm^{-2} (c) and 1.43 mA cm^{-2} (d).

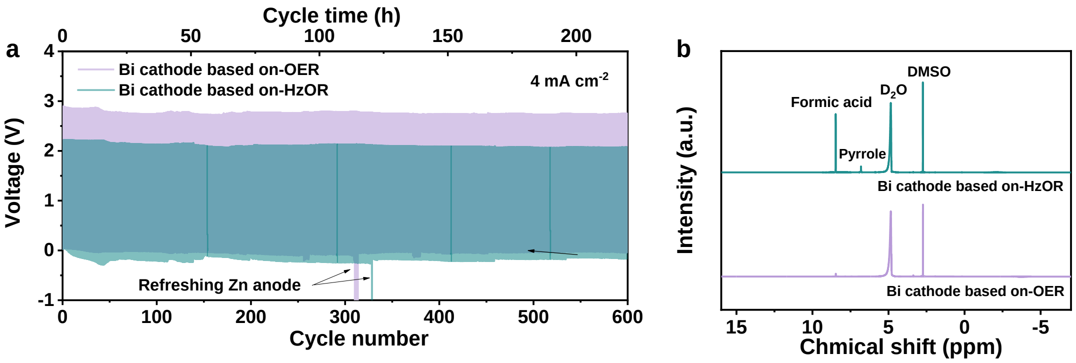

Supplementary Figure 17. Performance of the Zn-CRR/HzOR battery with Bi nanosheet as the catalyst for HCOOH generation. (a) Compared to the Zn-CO_2 battery with the OER reaction on charge, the Zn-CRR/HzOR battery shows a lower charging voltage. (b) The ^1H NMR spectrum apparently shows a dominant presence of formic acid for battery using HzOR compared to that using OER.

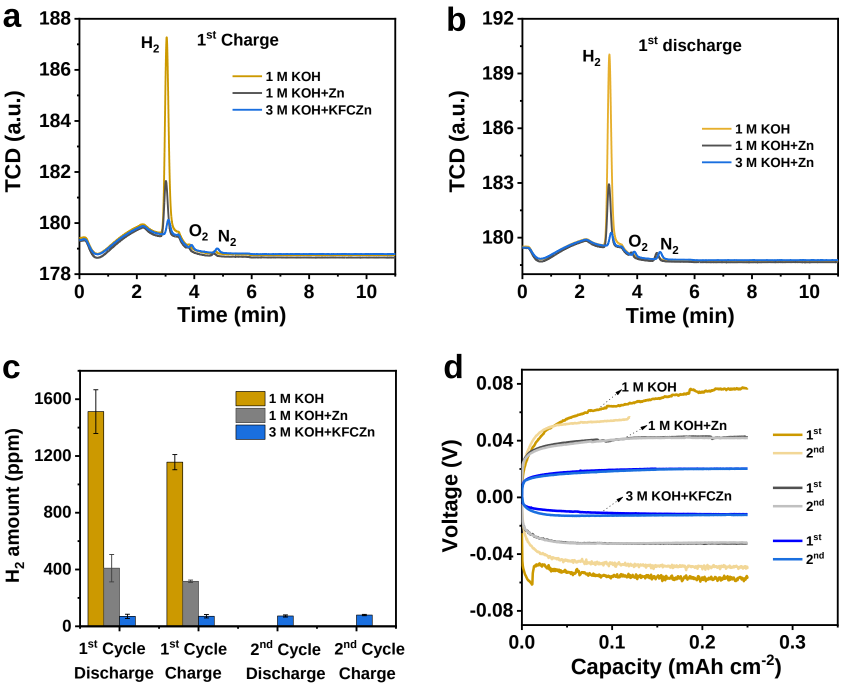

Supplementary Figure 18. Experimental measurements of hydrogen evolution in symmetric zinc batteries in different electrolytes. (a-b) The signals of thermal conductivity detector (TCD) during initial discharge (a) and charge (b) process demonstrating the inhibited HER in the newly formulated electrolyte compared to that in 1 M KOH + Zn. (c) The quantitation of H_2 generated during the cycling process by online GC, confirming the enhanced inhibition of HER in 3 M KOH + KFCZnO electrolytes. (d) Voltage profiles for Zn symmetric batteries during Zn stripping/plating at 0.33 mA cm^{-2}.

Supplementary Note 7: We first examined the HER behavior at the Zn anode by online

gas chromatography (GC), including 1 M KOH, 1 M KOH + Zn, 3 M KOH + KFCZn. Regarding the 3 M KOH + KFCZn electrolyte, in addition to quantifying H_2 amount in the first cycle, we also measured the second cycle for a further demonstration. H-type cells were used with zinc foil as both the cathodic and anodic electrodes. The zinc plating and stripping were performed at a current density of 0.33 mA cm^{-2}. Apparently, H_2 evolution occurs intensely in 1 M KOH as quantified by online GC in both discharge and charge processes (**Supplementary Figure 18a, b**). The average amount of H_2 generated in 3 M KOH + KFCZn electrolyte (65 ~ 75 ppm) is much lower than that in 1 M KOH + Zn (317 ~ 409 ppm) and 1 M KOH (1156 ~ 1512 ppm) (**Supplementary Figure 18c**). Thus, the HER activity trend of the Zn anodes in these electrolytes follows the order of 1 M KOH > 1 M KOH + Zn > 3 M KOH + KFCZn. The trend is also consistent with the magnitude of voltage polarization of the batteries (**Supplementary Figure 18d**), confirming the impact of HER on the polarization[52]. The results show that the original electrolyte (1 M KOH + Zn) shows a rather decent inhibiting effect on HER[52,53], and that our newly formulated electrolyte 3 M KOH + KFCZn further enhances the ability to inhibit the HER.

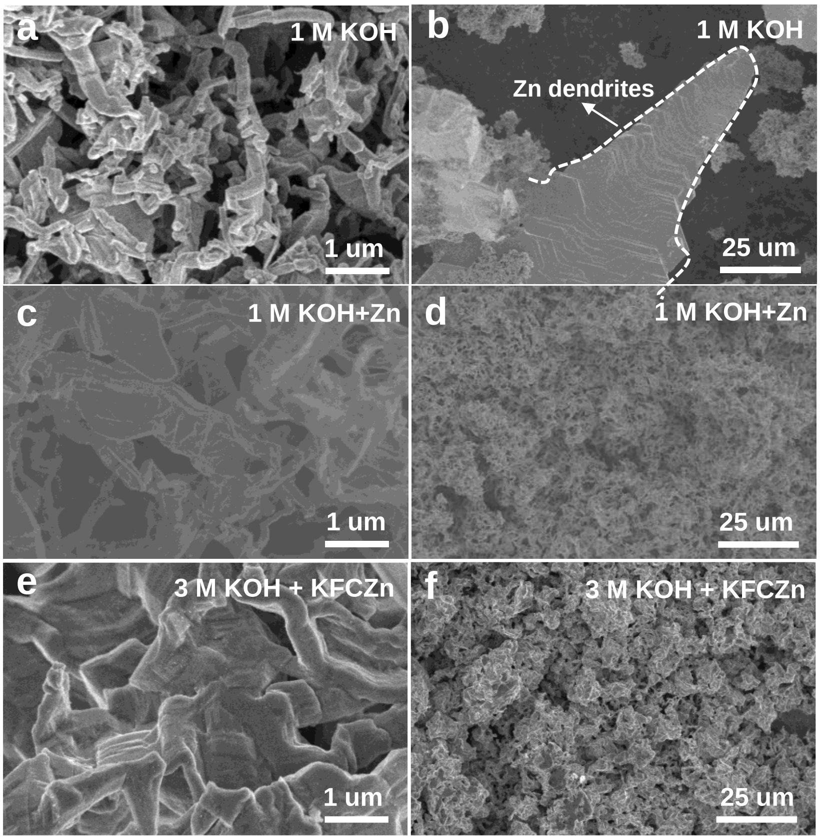

Supplementary Figure 19. SEM images of the Zn stripping/platting based on different electrolytes. (a-b) 1 M KOH; (c-d) 1 M KOH + Zn; (e-f) 3 M KOH + KFCZn.

Supplementary Note 8: We also studied the intrinsic behavior in the originally used 1 1 M KOH + Zn electrolyte with a plating capacity of 10 mAh cm^{-2}. Using a 1 M KOH blank electrolyte as a comparison, we observed very loose and uneven Zn surface with random appearance of obvious dendrites, reflecting a very uneven Zn plating process in 1 M KOH (**Supplementary Figure 19a, b**). Meanwhile, we did not observe distinct dendrites in the 1 M KOH + Zn electrolyte, as extensively observed for 1 M $ZnSO_4$, 6 M KOH + 0.2 M ZnO, 1 M $ZnSO_4$ in a mixture of DI H_2O and propylene glycol electrolytes in the literatures[54-56]. Instead, we observed formation of mossy fibrous deposits with a large size than those in the 1 M KOH blank electrolyte (**Supplementary Figure 19c, d**). This is probably due to the 0.02 M $Zn(CH_3COO)_2$ additive, wherein both Zn^{2+} and acetate anions can inhibit hydrogen generation and benefit the uniform Zn^{2+} plating process[57-58].

We improve the zinc anode by fundamentally altering its plating behavior through formulating functionalized electrolyte (3 M KOH + KFCZnO). The ZnO powder is added to inhibit the hydrogen evolution reaction, while the KF and K_2CO_3 are added to further suppress the dissolution and corrosion of Zn[59]. Indeed, with this

newly formulated electrolyte, the zinc was plated as a more uniform, and denser deposits, demonstrating efficient inhibition of Zn dendrites (**Supplementary Figure 19 e-f**). Furthermore, the dense and flat Zn plating layer indicates a much lower fraction of “dead” Zn after plating.

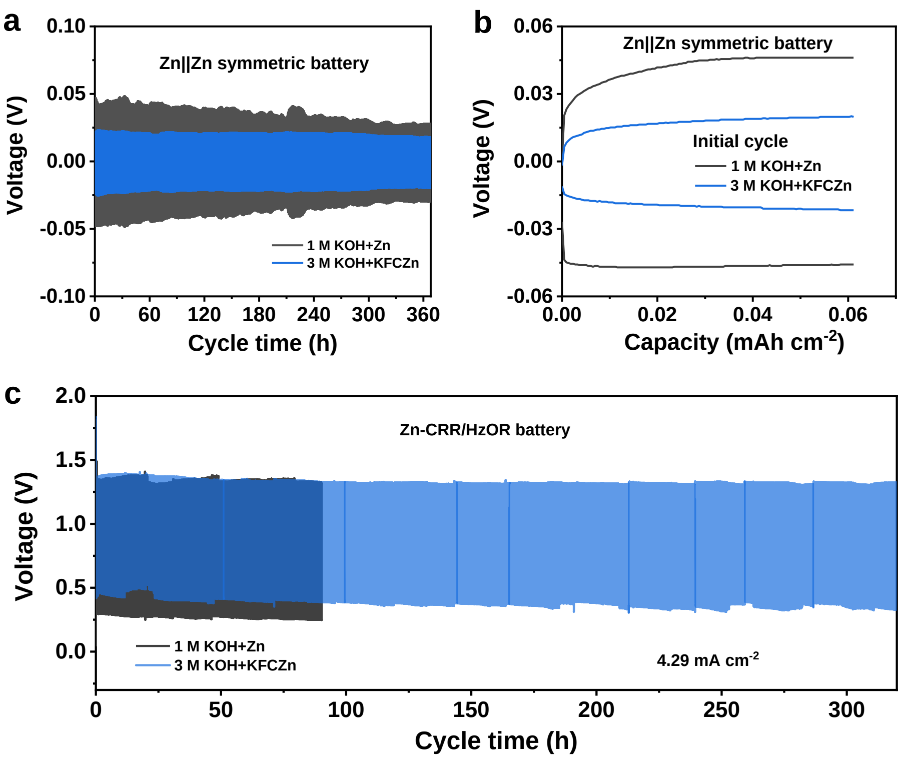

Supplementary Figure 20. Electrochemical performance of symmetric batteries of Zn electrodes and Zn-CRR/HzOR battery. (a) Long-term Zn stripping/plating cycling of symmetric batteries at 0.33 mA cm^{-2}. (b) Comparison of voltage profiles for Zn||Zn symmetric batteries in different electrolytes during Zn stripping/plating. (c) Higher discharging voltage obtained with 3 M KOH + KFCZnO anolyte (catholyte: 0.5 M $KHCO_3$ + 3.5 M KCl) compared to that using 1 M KOH + Zn anolyte (catholyte: 0.5 M $KHCO_3$). Notably, the 3 M KOH + KFCZnO anolyte mainly contributes to the increased voltage by the comparison of two catholytes (0.5 M $KHCO_3$ + 3.5 M KCl *vs*. 0.5 M $KHCO_3$).

Supplementary Note 9: We further measured the long-term stability of the Zn||Zn symmetric cells using various anolytes (**Supplementary Figure 20a**). Compared to the cell with the original 1 M KOH + Zn electrolyte, the symmetric cell with the 3 M KOH + KFCZnO electrolyte shows a lower voltage polarization likely due to the suppressed hydrogen evolution, less dead zinc and lower concentration polarization (**Supplementary Figure 20b**). Over long-term charge/discharge cycling at a current of 0.33 mA cm^{-2}, the cell shows stabilized polarization for over 360 hours. Notably, using

this newly formulated electrolyte as the anolyte, enhanced rate capabilities with stable cycling performance were obtained for the Zn-CRR/HzOR battery (**Supplementary Figure 20c**). Particularly, replacing 1 M KOH + Zn anolyte with the 3 M KOH + KFCZnO electrolyte, the discharged voltage was improved from 0.32 V to 0.44 V at a current of 4.29 mA cm^{-2}, and the battery maintains stable cycling for >320 hours, without need to refresh the zinc anode.

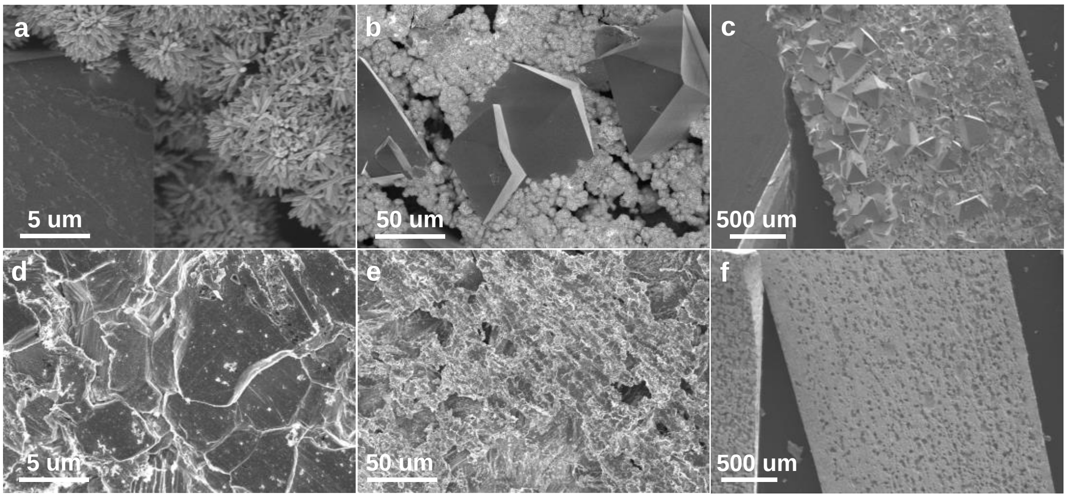

Supplementary Figure 21. The morphology of Zn anode in the Zn-CRR/HzOR battery after 450 cycles using the 1 M KOH + Zn (a-c) and 3 M KOH + KFCZn (d-f) electrolytes.

Supplementary Note 10: Compared to the case of 1 M KOH + Zn electrolyte, Zn stripping/platting was much more uniform and flatter after 450 cycles in the Zn-CRR/HzOR battery using the 3 M KOH + KFCZnO anolyte (**Supplementary Figure 21**), indicating its higher reversibility of Zn anode to realize the less often need for refreshing the zinc anode.

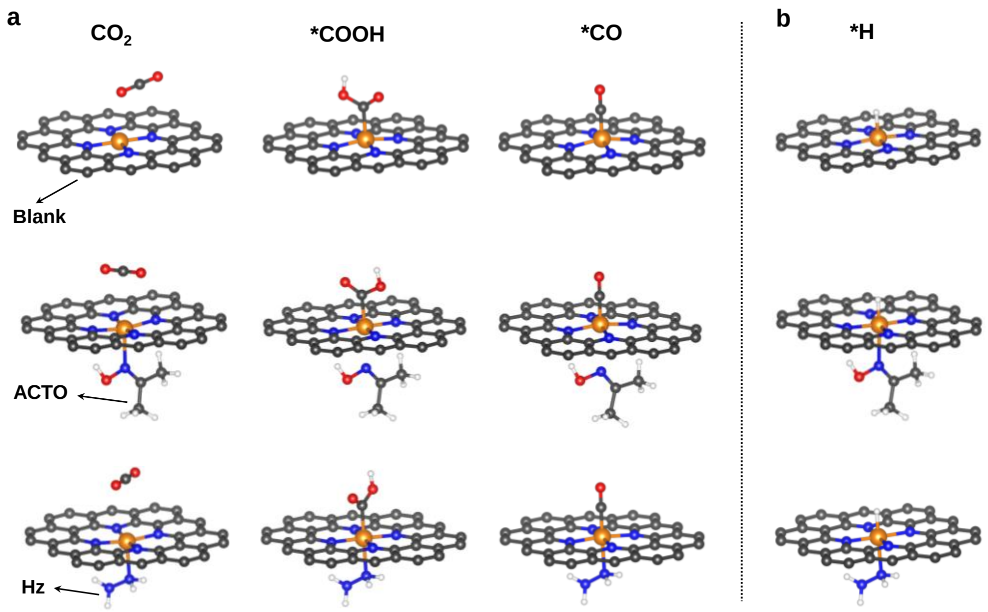

Supplementary Figure 22. Computational results of geometric configurations with reducing molecules adsorbing on the FeN_4 sites of a Fe-N-C catalyst. (a-b) The coordination of Fe active sites with ACTO and Hz molecules adsorbed on FeN_4 sites, which is different from that in blank FeN_4 sites during CRR (a) and HER (b) processes. The ACTO and Hz apparently interact with the Fe active sites and weaken the interaction of *CO with Fe, thus facilitating the desorption of CO. (Orange, blue, grey, red, and white balls stand for Fe, N, C, O, and H atoms).

Supplementary Note 11: A different projection of Fe-N-C catalyst with adsorbed reducing molecules during CRR and HER processes are provided in **Supplementary Figure 22**. This configuration (reducing molecules adsorbed on Fe atoms) suppresses H_2 generation at ACTO-FeN_4 sites owed to the highest energy barrier, in good accordance with our experimental observations on FE_{H_2}. In addition, we suspect that it may also be beneficial for the processes of ACTOR and HzOR because of the possible weak binding of Fe center with their electron-donating intermediates.

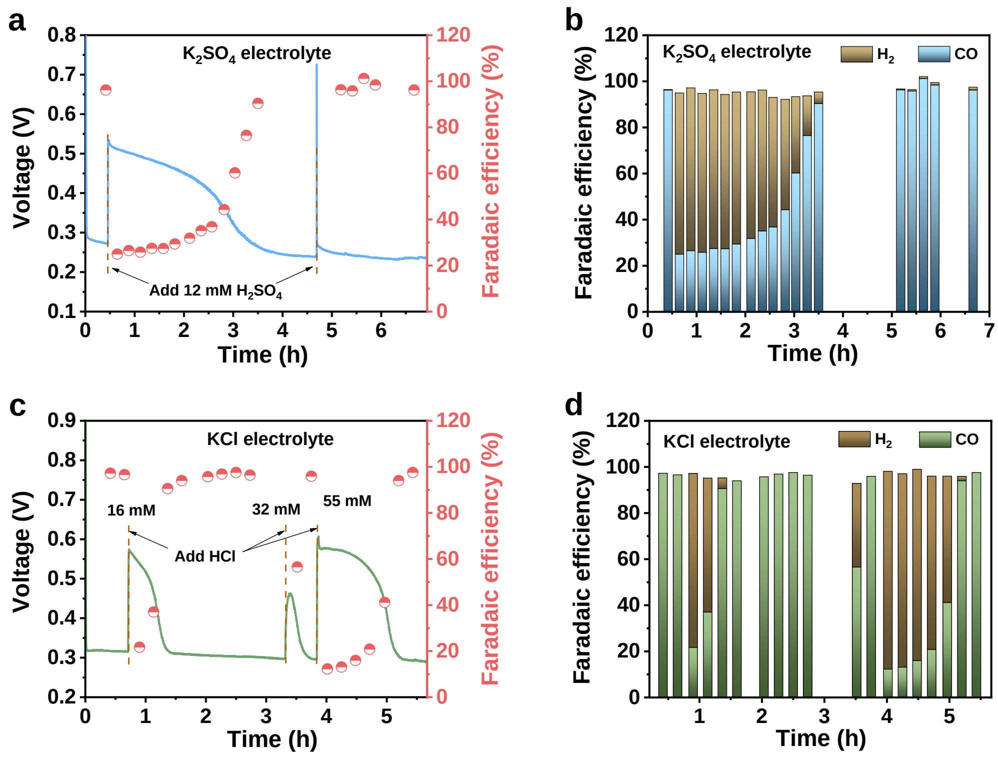

Supplementary Figure 23. The impact of presence of additional H^+ on the selectivity toward CO generation. (a-d) The variation of galvanostatic discharge profiles at 4.29 mA cm^{-2} and the Faradaic efficiency of CO were recorded after adding additional H^+ in two types of electrolytes including 0.5 M K_2SO_4 (a-b) and 0.5 M KCl (c-d). We observed an increase in voltage and decrease in Faradaic efficiency of CO after adding additional H^+, and the solutions gradually return back along with the consumption of H^+. Notably, the competitive advantages of HER over CO generation with the presence of extra H^+ is obvious.

Supplementary Note 12: To elucidate the intrinsic CRR activity in TUDO-based cathode, the proton-released experiments were first conducted. The results in **Supplementary Figure 23** clearly illustrate that the CRR activity was dramatically suppressed with the additional H^+, and further the FE_{CO} gradually increased and fully recovered with gradual consumption of H^+. These experiments confirm that the extra H^+ derived from TUDO is the cause of the decrease in FE_{CO}. It is noted that TUDO first decomposes into urea and H_2SO_3, and thus H_2SO_3 serves as the final reductant. Further details about the oxidation reaction process are presented in **Supplementary Figure 13-14**.

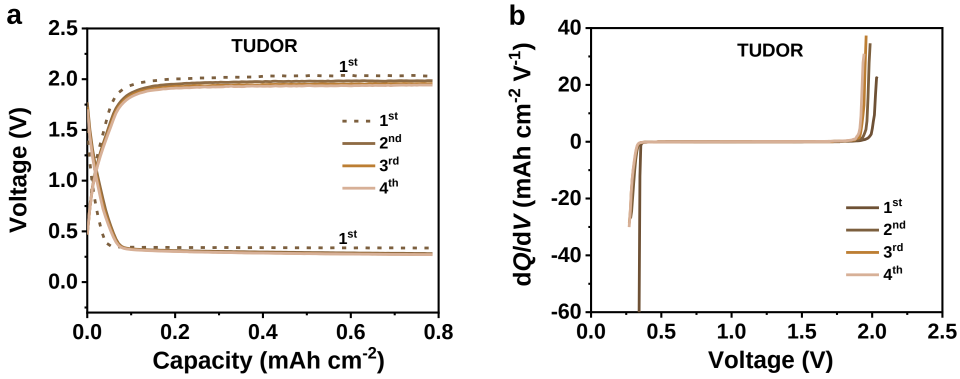

Supplementary Figure 24. Galvanostatic measurement of Zn-CRR/TUDOR battery. The initial four discharge/charge profiles (a) and corresponding dQ/dV profiles (b), indicating there is no Fe oxidation process occurring in the Zn-CRR/TUDOR battery.

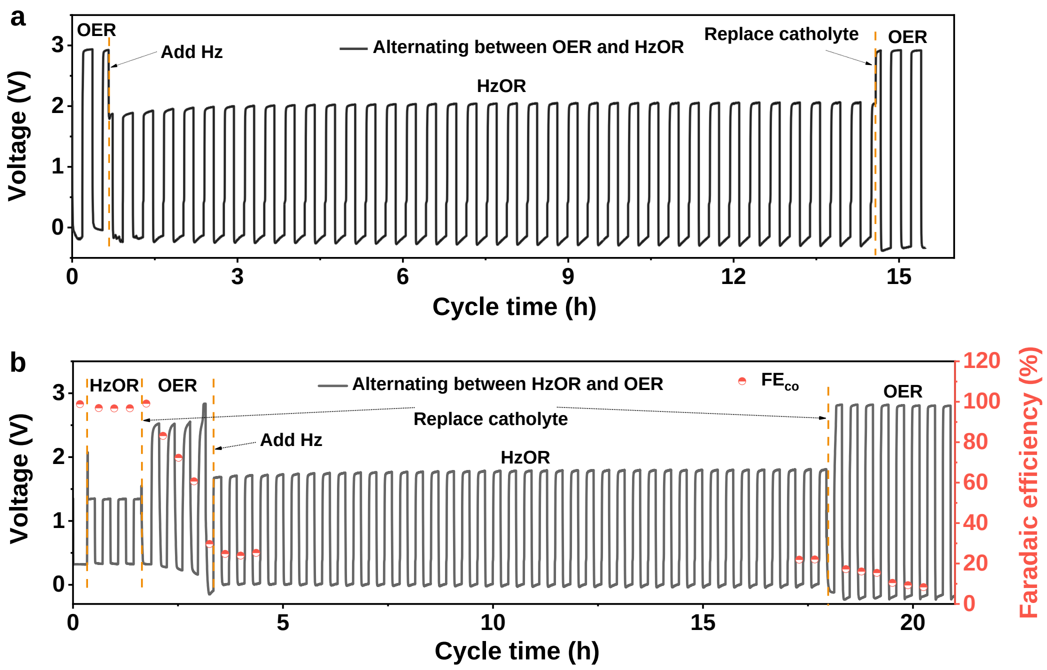

Supplementary Figure 25. Zn-CRR batteries operating by alternating the oxidation reactions between OER and HzOR. (a-b) Recharging the battery following the order of OER-HzOR-OER (a), or HzOR-OER-HzOR-OER (b). We observe that much higher voltage polarization always occurred once OER was involved. Furthermore, FE_{CO} was sharply reduced from 98% to 28% with OER (b), and it could be recovered even when HzOR was introduced back to the system, indicating that OER indeed caused the irreversible structural degradation.

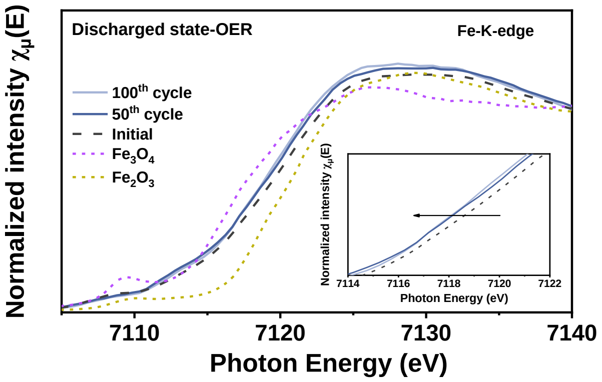

Supplementary Figure 26. Fe K-edge XANES spectra of OER-based electrode at the discharged state. The Fe K-edge spectra of cathode after 50 cycles almost coincides with that after 100 cycles, indicating similar Fe valence states when recharging the battery with OER over cycling.

Supplementary Note 13: For the Fe-N-C catalyst, it is known that Fe in the pyridinic N-coordinated (S1) sites tends to possess a +2 valence, and shows high activity but low structural stability (i.e., Fe demetalization). However, for the pyrrolic N-coordinated (S2) sites, Fe valence tends to be +3, and the S2 site generally shows lower catalytic activity but higher structural stability[60-62].

In our experiments, after cycling, the valence state of Fe in the OER-based cathode shifts to +3, indicating that the ratio of S2 site Fe^{3+} increases after Fe demetallation from S1 sites, leading to lower CRR activity in the OER-based cathode[60-62]. In contrast, the valence state of Fe in the HzOR-based cathode shifts to +2, implying an increased ratio of Fe^{2+} (S1 site) after Fe demetallation, thereby maintaining high selectivity for CO generation[60-62]. Fe demetallation was detected in both HzOR and OER based cathodes, as indicated by the ICP measurements discussed below (**Supplementary Tables 5-7**).

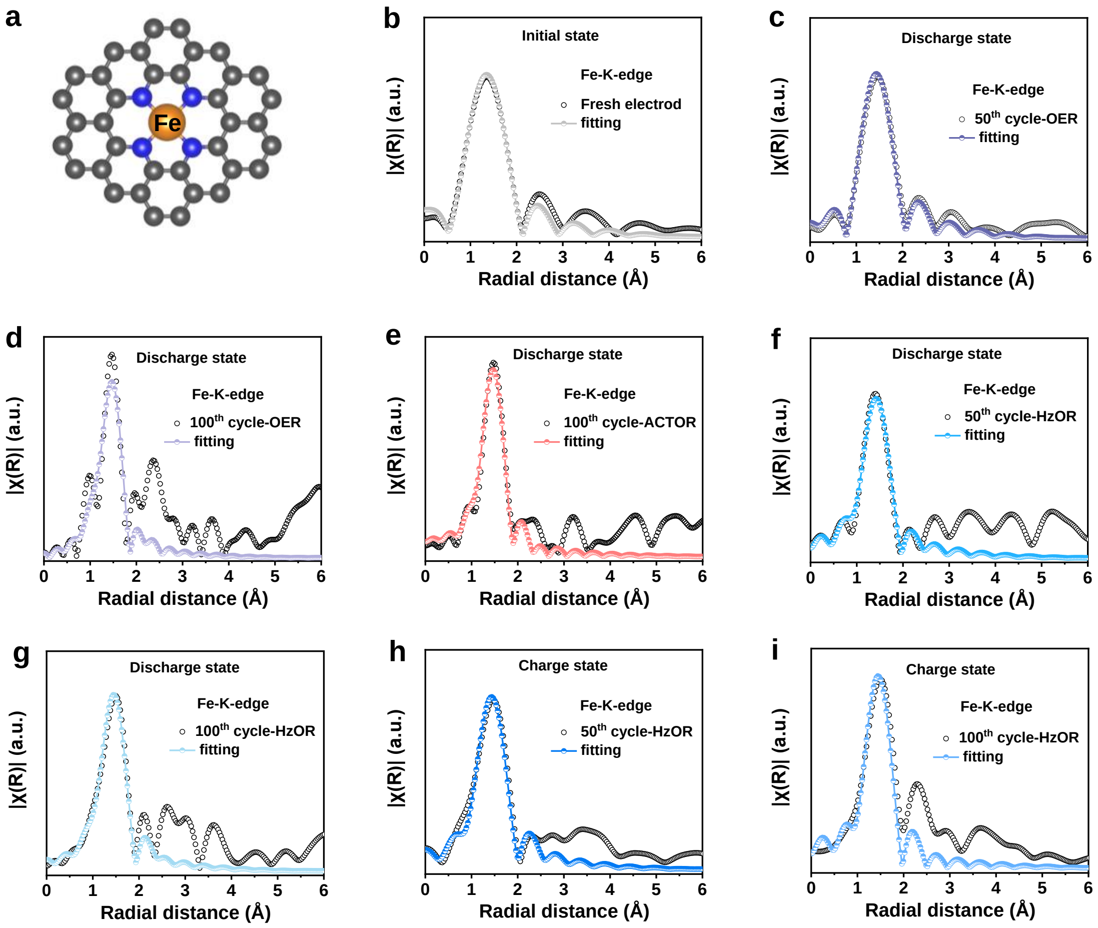

Supplementary Figure 27. Fitting results for the Fe K-edge EXAFS spectra of Fe-N-C cathodes at different states. Scheme of the Fe–N scattering paths (a) used to fit the first shell of Fe K-edge, and the EXAFS spectra fittings (b-h), which suggest that the FeN_4 configuration works well and can be considered the optimized configuration. The states of the Fe-N-C cathodes for the Fe K-edge EXAFS spectra are: (b) Initial state before cycling, (c, d) the discharged OER-based electrodes after 50 cycles (c) and 100 cycles (d), the discharged ACTOR-based electrode after 100 cycles (e), (f, g) the discharged HzOR-based electrodes after 50 cycles (f) and 100 cycles (g), and (h, i) the charged HzOR-based electrodes after 50 cycles (h) and 100 cycles (i). (The orange, blue, and gray spheres represent Fe, N, and C atoms, respectively)

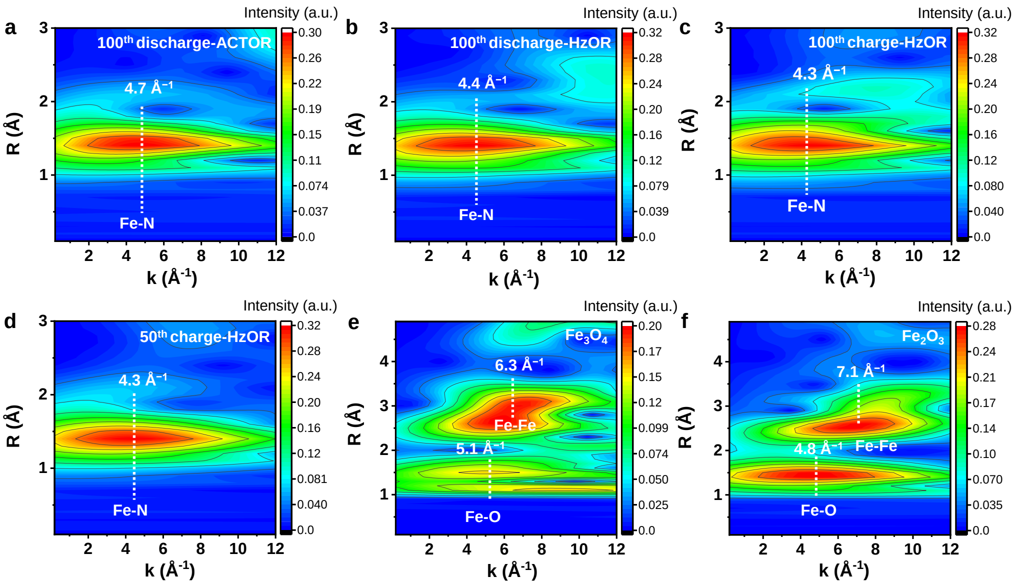

Supplementary Figure 28. Fe K-edge WT-EXAFS spectra for the cathodes with different oxidation reactions after cycling. The WT-EXAFS spectra of cathodes with ACTOR (a) and HzOR (b-d) oxidation reactions after 100 cycles. The states of the above Fe-N-C cathodes are: (a) the discharged ACTOR-based cathode after 100 cycles, (b, c) the discharged (b) and charged (c) HzOR-based cathodes after 100 cycles, and (d) the charged HzOR-based cathode after 50 cycles. The comparison of the WT-EXAFS spectra with those of the Fe_3O_4 (e) and Fe_2O_3 references (f) indicates a Fe-N bonding for the Fe coordination, instead of Fe-Fe bonding (Fe_3O_4 (e) and Fe_2O_3 references locate at higher wavenumbers than the Fe-N-C cathodes). Furthermore, in the case of the HzOR-based cathode, Fe-N bonding locates at very close or even lower wavenumbers than the references, indicating Fe is further immune to the formation of Fe-Fe bonding (*i.e.*, Fe oxidation) over cycling.

Supplementary Table 4. Parameters of the best Fe K-edge EXAFS fitting results for the Fe-N-C based cathodes at the initial state and discharged states.

Samples	State	Shell	CN	R (Å)	σ^2 (10^{-3}Å^2)	ΔE_0 (eV)	R-factor
Fresh electrode	Initial	Fe-N	4	1.949	0.007	0.042	0.008
OER-electrode-50 cycles	Discharged	Fe-N	4	1.980	0.004	0.073	0.020
OER-electrode-100 cycles	Discharged	Fe-N	4	1.950	0.008	0.042	0.020
ACTOR-electrode-100 cycles	Discharged	Fe-N	4	1.977	0.007	0.070	0.028
HzOR-electrode-50 cycles	Discharged	Fe-N	4	1.932	0.008	0.025	0.011
HzOR-electrode-100 cycles	Discharged	Fe-N	4	1.975	0.012	0.067	0.015
HzOR-electrode-50 cycles	Charged	Fe-N	4	1.972	0.012	0.064	0.021
HzOR-electrode-100 cycles	Charged	Fe-N	4	1.994	0.009	0.086	0.036

CN: coordination number;

R: distance between absorber and backscatter atoms;

σ^2: Debye-Waller factor to describe the variance in due to disorder (both lattice and thermal);

ΔE_0: threshold energy correction.

R-factor is used to evaluate the quality of the fitting, and a smaller value means better fitting result.

Supplementary Table 5. Fe ICP measurements of the electrolyte solutions after undergoing CRR, OER, ACTOR and TUDOR reactions with different test conditions (*i-t* or *v-t* test).

**Samples**	**Electrolytes**	**Concentration (mg L^{-1})**
Pure 0.5 M $KHCO_3$	0.5 M $KHCO_3$	0.15
CRR (*i-t* test, −0.4 V, for 237 h)	0.5 M $KHCO_3$	0.12
OER (*v-t* test, 4.29 mA cm^{-2})	0.5 M $KHCO_3$	0.25
HzOR (*v-t* test, 4.29 mA cm^{-2})	0.5 M $KHCO_3$	0.31
ACTOR (*v-t* test, 4.29 mA cm^{-2})	0.5 M $KHCO_3$	-
TUDOR (*v-t* test, 4.29 mA cm^{-2})	0.5 M $KHCO_3$	-

Supplementary Note 14: We found that Fe was also detected in the original 0.5 M electrolyte, and therefore this concentration (0.15 mg L^{-1}) was used as the benchmark to evaluate the dissolution of Fe. The iron concentration of over 0.15 mg L^{-1} in both electrolytes with OER and HzOR illustrates the existence of Fe demetallation.

Supplementary Table 6. Fe ICP results of the electrolyte solutions after OER or HzOR by LSV measurements.

**Samples**	**Electrolytes**	**Concentration (mg L^{-1})**
OER	1 M KOH	0.23
HzOR	1 M KOH	0.16

Supplementary Note 15: The upper potential of OER is 1.8 V and the current density is only ~ 30 mA cm^{-2} due to its poor electrochemical kinetics. The high concentration of Fe (0.23 mg L^{-1}) in the electrolyte solution with OER illustrates that Fe dementalization occurred rapidly at high over-potential. In contrast, HzOR shows a lower upper potential (only 0.8 V) with an ultrahigh current density over 250 mA cm^{-2} shows a lower degree of Fe demetalization, demonstrating that HzOR at a low overpotential is critical for maintaining the CRR activity on FeN_4 sites.

Supplementary Table 7. Fe ICP results of the Zn-CO_2 battery and Zn-CRR/RMOR batteries after cycling under different conditions.

Samples	Current and cycling life	Electrolytes	Concentration (mg L^{-1})
Zn-CO_2 battery with OER	4.29 mA cm^{-2}, 10 cycles	0.5 M $KHCO_3$	0.45
Zn-CRR/TUDOR battery	4.29 mA cm^{-2}, 240 cycles	0.5 M $KHCO_3$	0.30
Zn-CRR/ACTOR battery	4.29 mA cm^{-2}, 240 cycles	0.5 M $KHCO_3$	0.12
Zn-CRR/HzOR battery	1.43 mA cm^{-2}, 2768 cycles	0.5 M $KHCO_3$	0.22
Zn-CRR/HzOR battery	4.29 mA cm^{-2}, 1000 cycles	0.5 M $KHCO_3$	0.06
Zn-CRR/HzOR battery	7.14 mA cm^{-2}, 1003 cycles	0.5 M $KHCO_3$	0.23

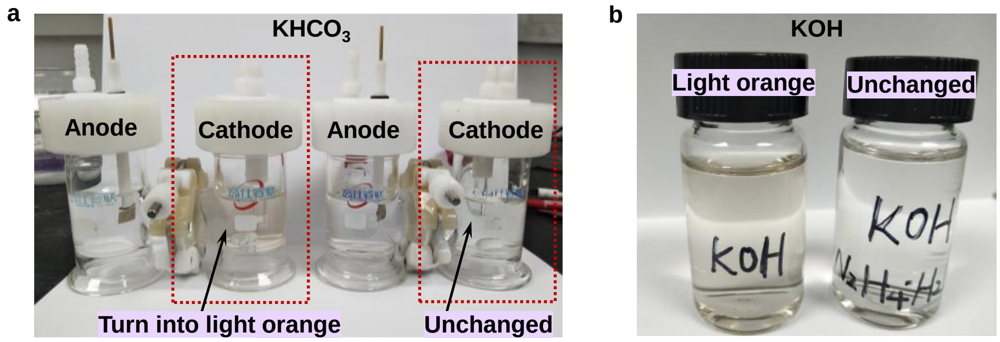

Supplementary Figure 29. The comparison of light orange electrolyte and the unchanged electrolyte after OER and HzOR. In (a, b), both electrolytes of 0.5 M $KHCO_3$ and 1 M KOH where OER occurs turn into light orange in contrast to the colorless one after HzOR.

Supplementary Note 16: A higher concentration of Fe was observed in the Zn-CO_2 battery with OER (0.45 mg L^{-1}) and with TUDOR (0.30 mg L^{-1}) compared to those with ACTOR and HzOR. This result implies that the activity decay in the Zn-CRR battery is attributed to the Fe demetallation from the FeN_4 moieties, eventually leading to the permanent loss of CRR activity.

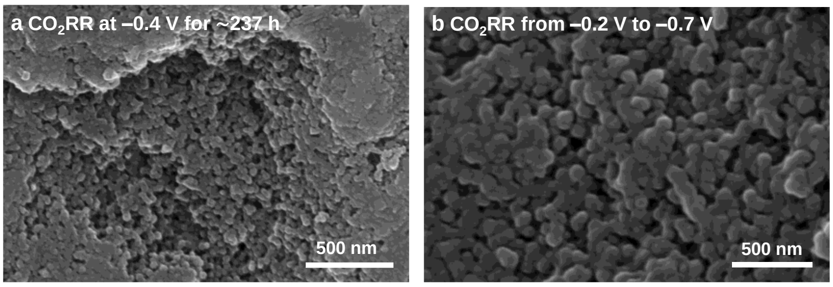

Supplementary Figure 30. SEM images of the Fe-N-C catalyst after CRR reactions. The nano-particles are still present after performing CRR at −0.4 V for 237 h (a) and at different potentials ranging from −0.2 V to −0.7 V (b), indicating that the morphological was well preserved after CRR test.

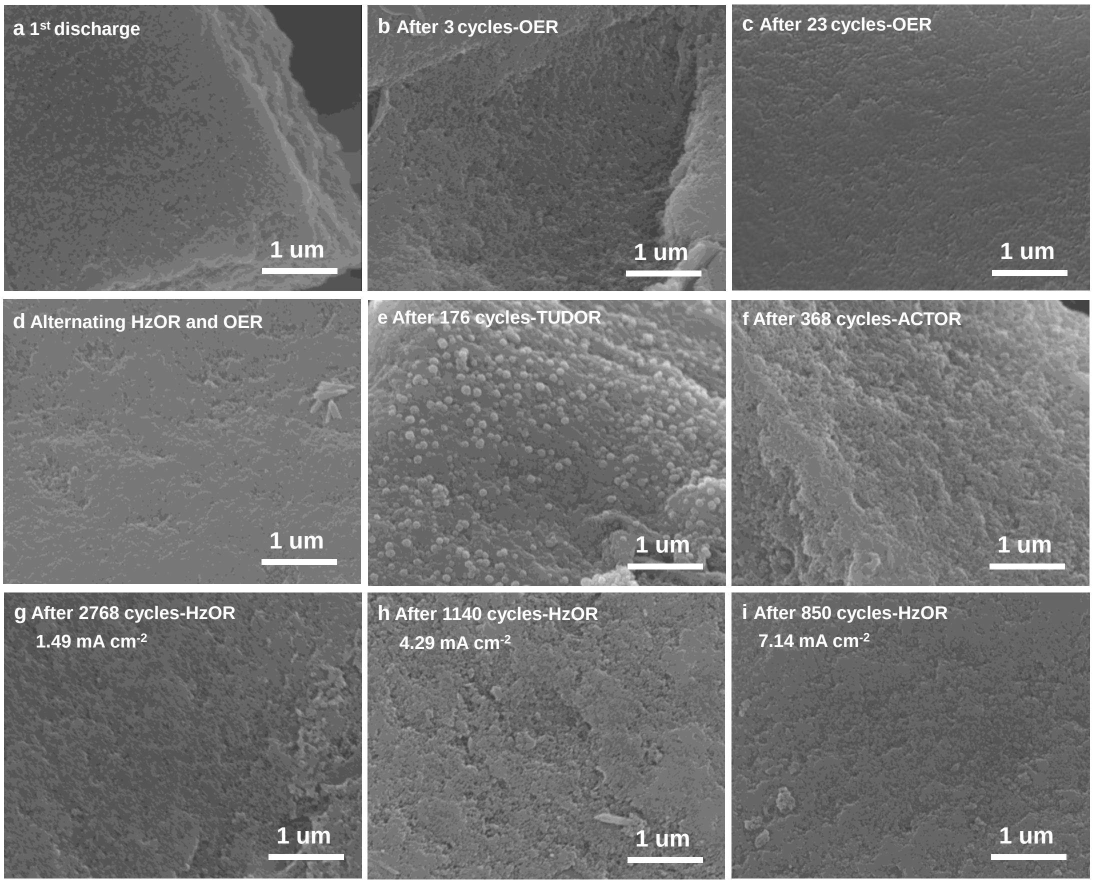

Supplementary Figure 31. Post-cycle SEM images of the Fe-N-C catalyst cathode based on different oxidation reactions. The SEM images of the cathodes after CRR (a), OER (b-c), alternating reactions between HzOR and OER (d), TUDOR (e), ACTOR (f), and HzOR (g-i) reactions, which show similar nano-particle morphology, indicating little change in morphological (carbon skeleton) after different oxidation reactions. The particles in (e) can be attributed to the potassium sulphate, which was derived from the TUDOR process.

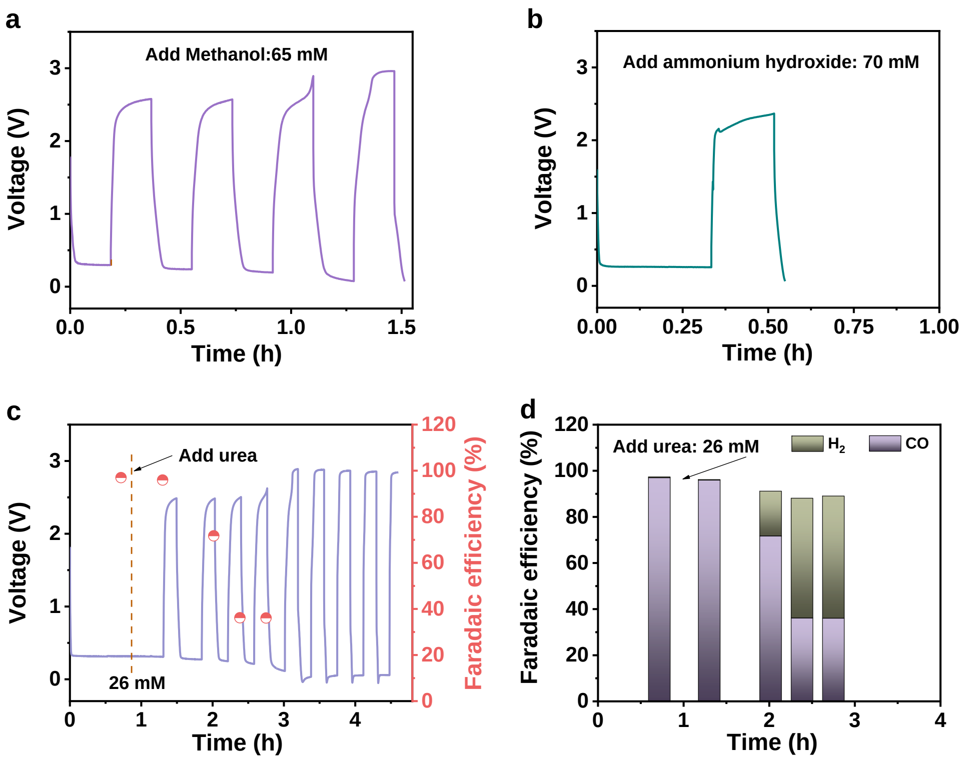

Supplementary Figure 32. The Zn-CRR/RMOR batteries based on the oxidation of non-reducing molecules. (a-c) Galvanostatic discharge-charge profiles of the batteries at 4.29 mA cm^{-2} with the presence of non-reducing molecules, including methanol (a), ammonium hydroxide (b), and urea (c), indicating the high charge voltage and limited cycling life. Moreover, a lower Faradaic efficiency of CO conversion was shown along with cycles after adding urea (c), which was accompanied by great H_2 generation (d), indicating that the non-reducing urea is not suitable for constructing the Zn-CRR/RMOR batteries.

Supplementary Note 17: In recent years, in the water splitting field, small organic molecules, such as methanol, ammonium hydroxide, urea have been developed as popular candidates to replace OER due to the lower overpotentials. However, they did not work out well when using as the charging active species in the Zn-CRR/RMOR batteries, as shown in **Supplementary Figure 32**, this is due to the absence of reducibility.

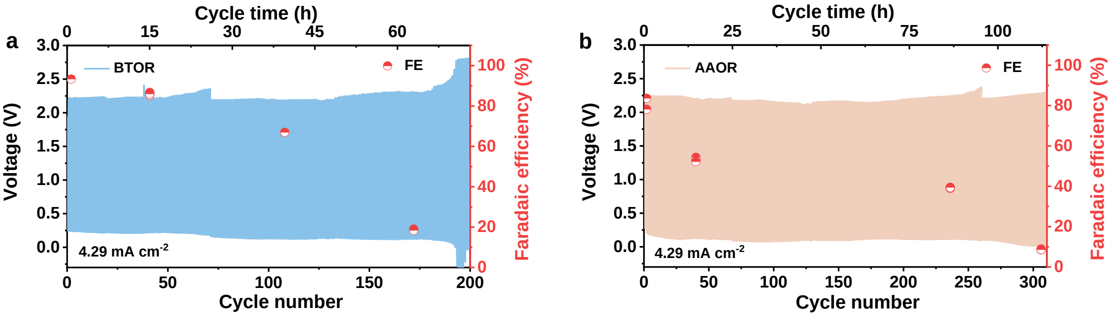

Supplementary Figure 33. The Zn-CRR/RMOR battery performance and the FE_{CO} based on another two reducing chemicals. BTO oxidation (BTOR) (a) and AAO oxidation (AAOR) (b) at 4.29 mA cm^{-2} further demonstrate that the strong reducibility is the prerequisite for the molecules to be used.

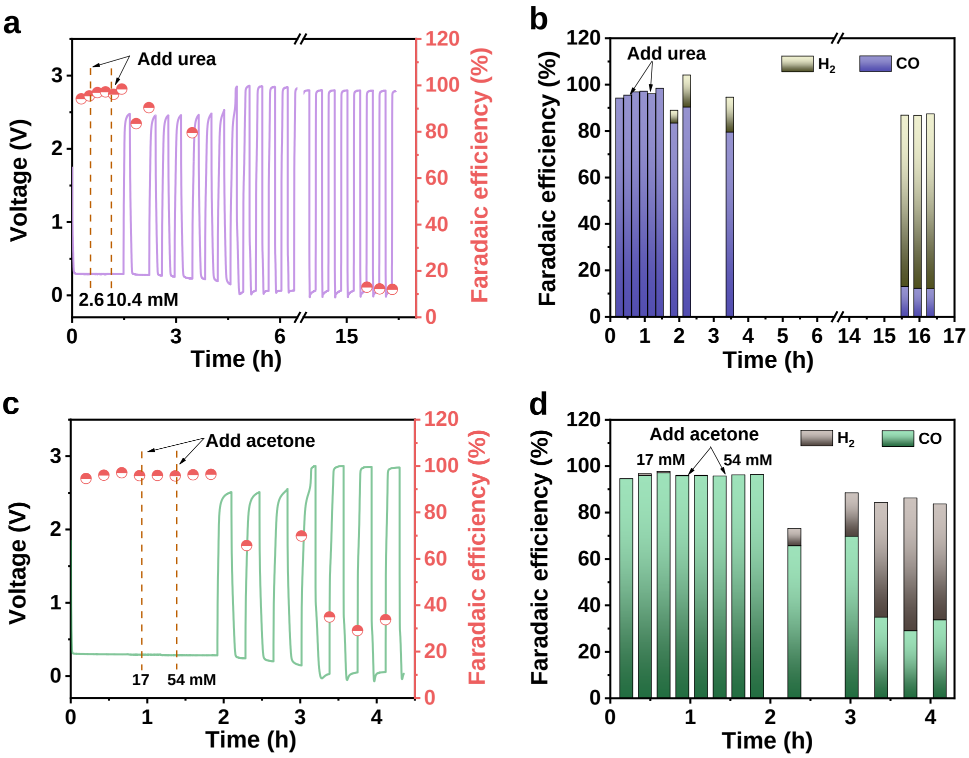

Supplementary Figure 34. Performance of the Zn-CRR/RMOR batteries based on the oxidation of urea and acetone. (a-d) Poor cycling performance of the Zn-CRR/RMOR batteries and low Faradaic efficiency of CO are shown after adding urea (a, b) and acetone (c, d) at 4.29 mA cm^{-2}, suggesting the organic oxidation product is undesired for the long-term cycling.

Supplementary Note 18: To understand the impact of the oxidation products (acetone and urea) on discharge and charge, we assembled the corresponding Zn-CRR/RMOR batteries. As shown in **Supplementary Figure 34**, the presence of both acetone and

urea has little impact on discharge process, while they cannot prolong the cycling life of battery because the high oxidation potential of charging reactions readily cause the structural degradation of Fe-N-C catalyst. Notably, the oxidation of urea was used to reveal the impact of TUDOR product on both the discharge and charge process.

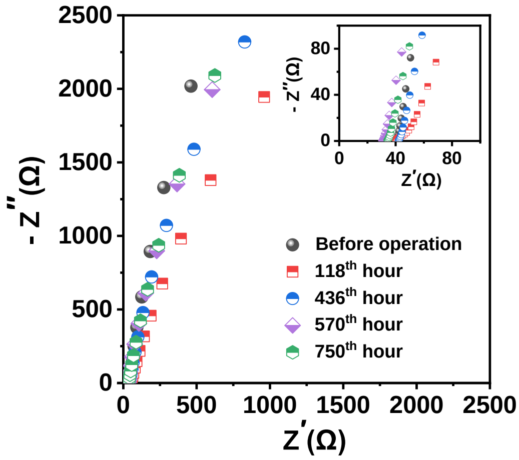

Supplementary Figure 35. The EIS results demonstrating similar R_s values after operations of 0, 118, 436, 570, and 750 hours.

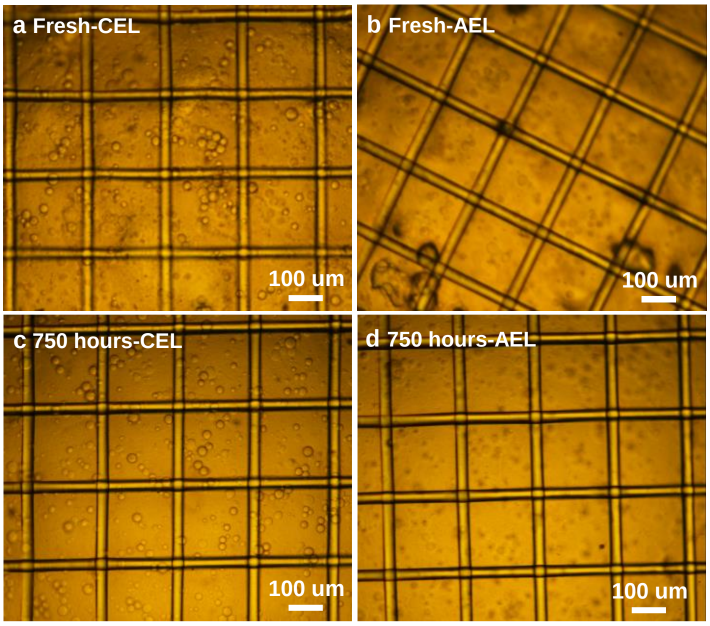

Supplementary Figure 36. Optical micrographs of the BPM before (a, b) and after 750 hours (c, d) showing no visible micro-tears or micro-cracks. Note that CEL and AEL are toward cathode and anode side, respectively.

Supplementary Note 19: We have now experimentally evaluated the stability of the

membrane by analyzing the impedance of the bipolar membrane after 750 hours' operation (2075 cycles) using the electrochemical impedance spectroscopy (EIS, **Supplementary Figure 35**) and optical microscope (**Supplementary Figure 36**). The solution contact impedance (R_s) is the primary indicator of the condition of the BPM to transport ions. As shown in **Supplementary Figure 35**, no obvious difference in the solution contact impedance values was observed, demonstrating the chemical integrity of BPM after long-term operations at 4.29 mA cm^{-2}. Further, we have now observed the microstructure of the membrane before and after 750 hour's operation by optical microscope, in order to confirm any microstructural damage that are invisible to the naked eye. As illustrated in **Supplementary Figure 36**, the results show that no micro-tears or micro-cracks are visible, proving its physical integrity. Therefore, the bipolar membrane is sufficiently stable for the battery operation of battery.

Using the anion exchange membrane (AEM), the impact of CRR performance of the Fe-N-C in cathode side can be neglected because the transferred OH^-, if any, will be rapidly consumed by HCO_3^- and CO_2. However, the crossover of HCO_3^- will hinder the stripping/plating of Zn anode as Zn dissolution prefers a basic solution. Over cycling, the degradation of Zn anode will lead to a significant reduction in both the discharged voltage and cycling life. The catholyte/anolyte stability challenge of crossover could be greatly alleviated by applying a bipolar membrane (BPM) that separately presents proton and hydroxyl to the two chambers.

References

1. Dhundhara, S., Verma, Y. P. & Williams, A. Techno-economic analysis of the lithium-ion and lead-acid battery in microgrid systems. *Energy Convers. Manage.* **177**, 122-142 (2018).
2. Lee, J. et al. Catholyte-free electroreduction of CO_2 for sustainable production of CO: concept, process development, techno-economic analysis, and CO_2 reduction assessment. *Green Chem.* **23**, 2397-2410 (2021).
3. Somoza-Tornos, A., Guerra, O. J., Crow, A. M., Smith, W. A. & Hodge, B.-M. Process

modeling, techno-economic assessment, and life cycle assessment of the electrochemical reduction of CO_2: a review. *iScience* **24**, 102813 (2021).

4. Feng, L., Tian, B., Wang, B. & Yang, M. Purification of hydrazine hydrate waste salt using a water washing based integrated process for the production of sodium hydrate via ion-exchange membrane electrolysis. *J. Cleaner Prod.* **319**, 128626 (2021).
5. Pan, F. et al. Unveiling active sites of CO_2 reduction on nitrogen-coordinated and atomically dispersed iron and cobalt catalysts. *ACS Catal.* **8**, 3116-3122 (2018).
6. Zhang, Y., Jiao, L., Yang, W., Xie, C. & Jiang, H. L. Rational fabrication of low-coordinate single-atom Ni electrocatalysts by MOFs for highly selective CO_2 reduction. *Angew. Chem. Int. Ed.* **60**, 7607-7611 (2021).
7. Yan, S. et al. Electron localization and lattice strain induced by surface lithium doping enable ampere-level electrosynthesis of formate from CO_2. *Angew. Chem. Int. Ed.* **60**, 25741-25745 (2021).
8. Zhang, Y. et al. Hierarchical cross-linked carbon aerogels with transition metal-nitrogen sites for highly efficient industrial-level CO_2 electroreduction. *Adv. Funct. Mater.* **31**, 2104377 (2021).
9. Gao, S. et al. Coralloid Au enables high-performance Zn–CO_2 battery and self-driven CO production. *J. Mater. Chem. A* **9**, 21024-21031 (2021).
10. Zeng, Z., Mohamed, A. G. A., Zhang, X. & Wang, Y. Wide potential CO_2-to-CO electroreduction relies on pyridinic-N/Ni–N_x sites and its Zn–CO_2 battery application. *Energy Technol.* **9**, 2100205 (2021).
11. Jiao, L. et al. Non-bonding interaction of neighboring Fe and Ni single-atom pairs on MOF-derived N-doped carbon for enhanced CO_2 electroreduction. *J. Am. Chem. Soc.* **143**, 19417-19424 (2021).
12. Zeng, Z. et al. Orbital coupling of hetero-diatomic nickel-iron site for bifunctional electrocatalysis of CO_2 reduction and oxygen evolution. *Nat. Commun.* **12,** 4088 (2021).
13. Ni, W. et al. Dual single-cobalt atom-based carbon electrocatalysts for efficient CO_2-to-syngas conversion with industrial current densities. *Appl. Catal., B* **291**, 120092 (2021).
14. Yang, M. et al. Highly dispersed Bi clusters for efficient rechargeable Zn−CO_2 batteries. *Appl. Catal., B* **307**, 121145 (2022).
15. Liu, S. et al. Coordination environment engineering to boost electrocatalytic CO_2 reduction performance by introducing boron into single-Fe-atomic catalyst. *Chem. Eng. J.* **437**, 135294 (2022).
16. Wang, Y. et al. Electron accumulation enables Bi efficient CO_2 reduction for formate production to boost clean Zn-CO_2 batteries. *Nano Energy* **92**, 106780 (2022).
17. Hao, J. et al. Strain relaxation in metal alloy catalysts steers the product selectivity of electrocatalytic CO_2 reduction. *ACS Nano* **16**, 3251-3263 (2022).

18. Zheng, W. et al. Atomically defined undercoordinated active sites for highly efficient CO_2 electroreduction. *Adv. Funct. Mater.* **30**, 1907658 (2019).

19. Gong, S. et al. Tuning the metal electronic structure of anchored cobalt phthalocyanine *via* dual-regulator for efficient CO_2 electroreduction and Zn–CO_2 batteries. *Adv. Funct. Mater.* **32**, 2110649 (2022).

20. Ni, W. et al. Electroreduction of carbon dioxide driven by the intrinsic defects in the carbon plane of a single Fe–N_4 site. *Adv. Mater.* **33**, 2003238 (2020).

21. Wang, T. et al. Gas diffusion strategy for inserting atomic iron sites into graphitized carbon supports for unusually high-efficient CO_2 electroreduction and high-performance Zn–CO_2 batteries. *Adv. Mater.* **32**, 2002430 (2020).

22. Wang, Y. et al. Bismuth with abundant defects for electrocatalytic CO_2 reduction and Zn–CO_2 batteries. *Chem. Commun*. **58**, 3621-3624 (2022).

23. Liu, S. et al. Nitrogen-doped carbon polyhedrons confined Fe–P nanocrystals as high-efficiency bifunctional catalysts for aqueous Zn−CO_2 batteries. *Small* **18**, 2104965 (2022).

24. Teng, X. et al. Selective CO_2 reduction to formate on a Zn-based electrocatalyst promoted by tellurium. *Chem. Mater.* **34**, 6036-6047 (2022).

25. Hao, J. et al. Interatomic electronegativity offset dictates selectivity when catalyzing the CO_2 reduction reaction. *Adv. Energy Mater.* **12**, 2200579 (2022).

26. Tan, Z. et al. BiO_{2-x} nanosheets with surface electron localizations for efficient electrocatalytic CO_2 reduction to formate. *CCS Chem.* **5**, 133-144 (2023).

27. Li, Q.-X. et al. Highly efficient electroreduction of CO_2 by defect single-atomic Ni-N_3 sites anchored on ordered micro-macroporous carbons. *Sci. China: Chem.* **65**, 1584-1593 (2022).

28. Peng, J.-X., Yang, W., Jia, Z., Jiao, L. & Jiang, H.-L. Axial coordination regulation of MOF-based single-atom Ni catalysts by halogen atoms for enhanced CO_2 electroreduction. *Nano Res.* **15**, 10063-10069 (2022).

29. Wang, X. et al. Rechargeable Zn–CO_2 electrochemical cells mimicking two-step photosynthesis. *Adv. Mater.* **31**, 1807807 (2019).

30. Li, Z. et al. Elucidation of the synergistic effect of dopants and vacancies on promoted selectivity for CO_2 electroreduction to formate. *Adv. Mater.* **33**, 2005113 (2020).

31. Yang, R. et al. A trifunctional Ni–N/P–O-codoped graphene electrocatalyst enables dual-model rechargeable Zn–CO_2/Zn–O_2 batteries. *J. Mater. Chem. A* **7**, 2575-2580 (2019).

32. Zhao, Y. et al. Rationally designed nitrogen-doped carbon macroporous fibers with loading of single cobalt sites for efficient aqueous Zn-CO_2 batteries. *Chem Catal.* **2**, 1480-1493 (2022).

33. Cao, X. et al. Atomic bridging structure of nickel–nitrogen–carbon for highly efficient electrocatalytic reduction of CO_2. *Angew. Chem., Int. Ed.* **61**, 202113918 (2021).

34. Chen, J. et al. Promoting CO_2 electroreduction kinetics on atomically dispersed monovalent Zn^{I} sites by rationally engineering proton-feeding centers. *Angew. Chem. Int. Ed.* **61**,

202111683 (2021).
35. Wang, J. et al. Tailoring the interactions of heterostructured $Ni_4N/Ni_3ZnC_{0.7}$ for efficient CO_2 electroreduction. *J. Energy Chem.* **75**, 1-7 (2022).
36. Zheng, W. et al. A universal principle to accurately synthesize atomically dispersed metal–N_4 sites for CO_2 electroreduction. *Nano-Micro Lett.* **12**, 108 (2020).
37. Dong, J. et al. Abundant (110) facets on $PdCu_3$ alloy promote electrochemical conversion of CO_2 to CO. *ACS Appl. Nano Mater.* **14**, 41969-41977 (2022).
38. Gao, S. et al. Bifunctional BiPd alloy particles anchored on carbon matrix for reversible Zn–CO_2 battery. *ACS Appl. Nano Mater.* **5**, 12387-12394 (2022).
39. Hu, X. et al. Boosting industrial-level CO_2 electroreduction of N-doped carbon nanofibers with confined tin-nitrogen active sites *via* accelerating proton transport kinetics. *Adv. Funct. Mater.* **33**, 2208781 (2022).
40. Kaur, S. et al. Efficient CO_2 utilization and sustainable energy conversion *via* aqueous Zn-CO_2 batteries. *Nano Energy* **109**, 108242 (2023).
41. Han, L. et al. Chemically coupling SnO_2 quantum dots and MXene for efficient CO_2 electroreduction to formate and Zn–CO_2 battery. *PNAS* **119**, 2207326119 (2022).
42. Zhang, M. et al. Few-atom-layer metallene quantum dots toward CO_2 electroreduction at ampere-level current density and Zn-CO_2 battery. *Chem Catal.* **2**, 3528-3545 (2022).
43. Liu, X., Wang, X., Yang, B., Zhang, J. & Lu, J. High-entropy layered oxides nanosheets for highly efficient photoelectrocatalytic reduction of CO_2 and application research. *Nano Res*. **16**, 4775–4785 (2023).
44. Wang, F. et al. Ultrathin nitrogen-doped carbon encapsulated Ni nanoparticles for highly efficient electrochemical CO_2 reduction and aqueous Zn-CO_2 batteries. *Small*, 2301128, (2023).
45. Xu, A. et al. Regulating the electronic structure of bismuth nanosheets by titanium doping to boost CO_2 electroreduction and Zn–CO_2 batteries. *Small*, 2302253 (2023).
46. Tian, H., Shui, Z., Raza, M. A., Zhu, L. & Chen, X. Synergistic catalysis of bimetallic nitrogen-doped carbon materials for efficient electrocatalytic CO_2 reduction. *J. Alloys Compd.* **958**, 170544 (2023).
47. Li, J. *et al.* Asymmetric coordinated single-atom Pd sites for high performance CO_2 electroreduction and Zn–CO_2 battery. *Chem. Eng. J.* **461**, 141865 (2023).
48. Cao, C. et al. Si doping-induced electronic structure regulation of single-atom Fe sites for boosted CO_2 electroreduction at low overpotentials. *Research* **6**, 0079 (2023).
49. Yang, W. et al. Fe nanoparticles embedded in N-doped porous carbon for enhanced electrocatalytic CO_2 reduction and Zn-CO_2 battery. *Chin. J. Catal.* **48**, 185-194 (2023).
50. Martín, A. J., Mitchell, S., Mondelli, C., Jaydev, S. & Pérez-Ramírez, J. Unifying views on catalyst deactivation. *Nat. Catal.* **5**, 854-866 (2022).
51. Liu, S. et al. Atomically dispersed iron sites with a nitrogen–carbon coating as highly active

and durable oxygen reduction catalysts for fuel cells. *Nat. Energy* **7**, 652-663 (2022).

52. Hao, J., Yuan, L., Zhu, Y., Jaroniec, M. & Qiao, S. Z. Triple-function electrolyte regulation toward advanced aqueous Zn-ion batteries. *Adv. Mater.* **34**, 2206963 (2022).
53. Chen, S. et al. An asymmetric electrolyte to simultaneously meet contradictory requirements of anode and cathode. *Nat. Commun.* **14**, 2925 (2023).
54. Li, Y. et al. A progressive nucleation mechanism enables stable zinc stripping–plating behavior. *Energy Environ. Sci.* **14**, 5563-5571 (2021).
55. Liang, P. et al. The magnetohydrodynamic effect enables a dendrite-free Zn anode in alkaline electrolytes. *J. Mater. Chem. A* **10**, 11971-11979 (2022).
56. Shang, Y. et al. Long-life Zn anode enabled by low volume concentration of a benign electrolyte additive. *Adv. Funct. Mater.* **32**, 2200606 (2022).
57. Yang, M., Zhu, J., Bi, S., Wang, R. & Niu, Z. A binary hydrate-melt electrolyte with acetate-oriented cross-linking solvation shells for stable zinc anodes. *Adv. Mater.* **34**, 2201744 (2022).
58. Gomez Vazquez, D. et al. Creating water-in-salt-like environment using coordinating anions in non-concentrated aqueous electrolytes for efficient Zn batteries. *Energy Environ. Sci.* **16**, 1982-1991 (2023).
59. Zhou, W. et al. A scalable top-down strategy toward practical metrics of Ni–Zn aqueous batteries with total energy densities of 165 W h kg^{-1} and 506 W h L^{-1}. *Energy Environ. Sci.* **13**, 4157-4167 (2020).
60. Li, J. et al. Identification of durable and non-durable FeN_x sites in Fe–N–C materials for proton exchange membrane fuel cells. *Nat. Catal.* **4**, 10-19 (2020).
61. Gu, J., Hsu, C.-S., Bai, L., Chen, H. M. & Hu, X. Atomically dispersed Fe^{3+} sites catalyze efficient CO_2 electroreduction to CO. *Science* **364**, 1091-1094 (2019).
62. Martín, A. J., Mitchell, S., Mondelli, C., Jaydev, S. & Pérez-Ramírez, J. Unifying views on catalyst deactivation. *Nature Catal.* **5**, 854-866 (2022).
